# Sugar Concentration, Nitrogen Availability, and Phylogenetic Factors Determine the Ability of *Acinetobacter* spp. and *Rosenbergiella* spp. to Grow in Floral Nectar

**DOI:** 10.1007/s00248-022-02088-4

**Published:** 2022-08-05

**Authors:** José R. Morales-Poole, Clara de Vega, Kaoru Tsuji, Hans Jacquemyn, Robert R. Junker, Carlos M. Herrera, Chris Michiels, Bart Lievens, Sergio Álvarez-Pérez

**Affiliations:** 1 Department of Animal Health, Complutense University of Madrid, 28040 Madrid, Spain; 2 Departamento de Biología Vegetal y Ecología, Universidad de Sevilla, 41012 Seville, Spain; 3 Department of Biology, Graduate School of Science, Kobe University, Hyogo, 657-8501 Japan; 4 Laboratory of Plant Conservation and Population Biology, Biology Department, KU Leuven, B-3001 Heverlee, Belgium; 5 Evolutionary Ecology of Plants, Department of Biology, Philipps-University Marburg, 35043 Marburg, Germany; 6 Department of Biosciences, University Salzburg, 5020 Salzburg, Austria; 7 Estación Biológica de Doñana, CSIC, 41092 Seville, Spain; 8 Laboratory of Food Microbiology, Department of Microbial and Molecular Systems, KU Leuven, B-3001 Heverlee, Belgium; 9 Laboratory for Process Microbial Ecology and Bioinspirational Management (PME&BIM), Department of Microbial and Molecular Systems, KU Leuven, B-3001 Heverlee, Belgium

**Keywords:** *Acinetobacter*, Floral nectar, Bee, Phylogeny, *Rosenbergiella*

## Abstract

The floral nectar of angiosperms harbors a variety of microorganisms that depend predominantly on animal visitors for their dispersal. Although some members of the genus *Acinetobacter* and all currently known species of *Rosenbergiella* are thought to be adapted to thrive in nectar, there is limited information about the response of these bacteria to variation in the chemical characteristics of floral nectar. We investigated the growth performance of a diverse collection of *Acinetobacter* (*n* = 43) and *Rosenbergiella* (*n* = 45) isolates obtained from floral nectar and the digestive tract of flower-visiting bees in a set of 12 artificial nectars differing in sugar content (15% w/v or 50% w/v), nitrogen content (3.48/1.67 ppm or 348/167 ppm of total nitrogen/amino nitrogen), and sugar composition (only sucrose, 1/3 sucrose + 1/3 glucose + 1/3 fructose, or 1/2 glucose + 1/2 fructose). Growth was only observed in four of the 12 artificial nectars. Those containing elevated sugar concentration (50% w/v) and low nitrogen content (3.48/1.67 ppm) were limiting for bacterial growth. Furthermore, phylogenetic analyses revealed that the ability of the bacteria to grow in different types of nectar is highly conserved between closely related isolates and genotypes, but this conservatism rapidly vanishes deeper in phylogeny. Overall, these results demonstrate that the ability of *Acinetobacter* spp. and *Rosenbergiella* spp. to grow in floral nectar largely depends on nectar chemistry and bacterial phylogeny.

**Supplementary Information:**

The online version contains supplementary material available at 10.1007/s00248-022-02088-4.

## Introduction

Microbial growth in natural habitats largely depends on local environmental conditions, including diverse physico-chemical factors and the availability of key nutrients [1]. In host-associated microorganisms, such local environmental conditions can vary across potential hosts or even among different parts of the same host, so that each microhabitat may select for microbial populations displaying specific phenotypes and/or genotypes depending on the prevailing growth constraints and associated selective regime [2, 3]. Additionally, many microbial traits seem to be phylogenetically conserved, so that closely related taxa display more similar trait values than distant relatives do [4].

Land plants are hosts of diverse microbial communities, and specifically, the flowers of angiosperms provide diverse ephemeral habitats for microbial growth [5], of which floral nectar has received much attention in recent years. While floral nectar is assumed to be initially sterile, it often becomes rapidly colonized after anthesis by microorganisms (particularly yeasts and bacteria) associated with pollinators or other flower-visiting animals [6–8]. However, the typical chemical properties of floral nectar impose strong constraints for microbial life in this habitat [8–13]. In general, floral nectar is characterized by moderate to high sugar concentrations, which may exert high osmotic pressure, and low concentrations of other substances essential for microbial growth, such as amino acids and other nitrogen sources [11, 14–17].

Previous research has documented the presence of phylogenetically diverse bacteria in the floral nectar of diverse plant species worldwide [6, 18–25]. Members of the genera *Acinetobacter* and *Rosenbergiella* (*Gammaproteobacteria*) rank among the most frequent nectar inhabitants [6, 8, 9, 18, 26]. Furthermore, these two genera have been found in the mouthparts and digestive tract of diverse pollinators and other flower visitors, including insects (e.g., honeybees, bumblebees, and beetles) and hummingbirds [25, 27–29]. Although floral nectar is a complex solution of several nutrients, most of the research on microbial growth in nectar has focused on individual components (e.g., only sugars or only nitrogen sources). In this regard, it has been demonstrated that some *Acinetobacter* species and all *Rosenbergiella* species can tolerate high sugar concentrations (up to 60% w/v) and feed on different carbon and nitrogen sources [27, 30–36]. However, the combined effect of different chemical characteristics of nectar on the growth of nectar-inhabiting bacteria remains largely unexplored. Moreover, although phylogenetic affiliation seems to account for some variability in the growth on different nutrient sources of *Acinetobacter* species, so that closely related lineages (species or isolates) perform more similarly than do distantly related lineages [32, 36], it has not been determined yet if this factor has some influence on the growth of *Acinetobacter* and other bacterial taxa in floral nectar.

In this study, we investigated the growth performance of a diverse collection of *Acinetobacter* and *Rosenbergiella* isolates obtained from floral nectar and bees from different geographical origins in a set of twelve artificial nectars differing in their basic properties, namely, overall sugar and nitrogen content and sugar composition. Growth profiles were compared under the hypotheses that (i) under the combination of elevated sugar and low nitrogen content, the growth of *Acinetobacter* and *Rosenbergiella* isolates in nectar is limited, and (ii) the growth ability of these bacteria in different nectar types is determined by their phylogenetic affiliation.

## Materials and Methods

### Isolates

Forty-three *Acinetobacter* isolates and 45 *Rosenbergiella* isolates were used in this study (Tables S1 and S2, respectively). Studied isolates had been obtained between 2011 and 2018 from flowers and bees collected in different locations of Europe (Belgium, France, and Spain), the USA (California and Hawaii Island), South Africa, and Japan. Each of the isolates came from separate flowers or bees. All bacterial isolates analyzed in this study were grown on trypticase soy agar (TSA; Merck Life Science, Overijse, Belgium) at 25 °C and stored at − 80 °C in brain heart infusion (BHI) broth (Becton Dickinson, Erembodegem, Belgium) containing 25% glycerol (Merck Life Science) until further use.

Species-level classification of isolates was achieved by analyzing the sequence of a number of housekeeping genes commonly used as taxonomic markers of the *Gammaproteobacteria*, including *atpD*, which encodes the ATP synthase β-chain; *gyrB*, encoding the DNA gyrase subunit B; and/or *rpoB*, encoding the β subunit of RNA polymerase (see Supplementary Methods). Previous work has revealed that these housekeeping genes offer greater resolution than the 16S ribosomal RNA (rRNA) gene for discriminating between the *Acinetobacter* lineages (*rpoB*) and the *Rosenbergiella* lineages (*atpD*, *gyrB*, and *rpoB*) typically found in floral nectar and insects [30, 31, 33, 35].

*Acinetobacter* isolates included representatives of *A. nectaris* (*n* = 35; 81.4% of the total number of isolates of this genus) and *A. boissieri* (*n* = 8; 18.6%), which earlier studies have identified as the most prevalent species of the genus in floral nectar [18, 20, 23, 29] (Table S1). Ten *A. nectaris* isolates were obtained from the mouthparts, honey crop, or gut of honeybees (*Apis mellifera*) collected in Stanford campus (California, USA), whereas the remaining 25 *A. nectaris* isolates and all *A. boissieri* isolates had been retrieved from floral nectar of 11 plant species from nine families collected in Belgium (13 *A. nectaris* isolates from 2 plant species), Spain (5 *A. nectaris* isolates from 4 plant species and 8 *A. boissieri* isolates from 6 plant species), and the USA (7 *A. nectaris* isolates from *Epilobium canum* (Onagraceae)) (Table S1).

Most *Rosenbergiella* isolates (*n* = 41, 91.1% of the total number of isolates of this genus) had been obtained from floral nectar of diverse plant species (15 plant species from 12 families) collected in different countries, but four isolates (8.9%) had been retrieved from bees collected in Stanford Campus (California, USA) (Table S2). *Rosenbergiella* isolates included all validated species of the genus, namely *R. epipactidis* (*n* = 16 isolates (35.6%): 1 from France, 1 from Japan, 5 from Spain, and 9 from the USA, all of them from floral nectar), *R. nectarea* (*n* = 16 isolates (35.6%): 6 nectar isolates from Belgium, 2 nectar isolates from France, and 4 nectar isolates from the USA, plus 4 isolates from the mouthparts of honeybees (3 isolates) or the gut of a bumblebee (*Bombus* sp., 1 isolate) collected in the USA), *R. collisarenosi* (*n* = 6 isolates (13.3%): 2 from Belgium, 3 from Spain, and 1 from the USA, all of them from floral nectar), and *R. australiborealis* (*n* = 3 isolates (6.7%) found in nectar samples from South Africa) (Table S2). Additionally, 3 nectar isolates from Spain (6.7%) and one nectar isolate from Hawaii (USA, 2.2%) belonged to “*R. gaditana*” and “*R. metrosideri*,” respectively, two new *Rosenbergiella* species which are pending formal recognition [30].

### Growth in Artificial Nectars

All isolates were tested for their ability to grow in twelve types of artificial nectar. These nectars varied in total sugar concentration (15% or 50% w/v, coded as “s” and “S,” respectively; i.e., lowercase for low sugar level and uppercase for high sugar level), the availability of nitrogen sources (3.48/1.67 ppm and 348/167 ppm of total nitrogen/amino nitrogen; “n” and “N,” respectively; i.e., lowercase for low nitrogen level and uppercase for high nitrogen level), and their sugar composition (only sucrose, 1/3 sucrose + 1/3 glucose + 1/3 fructose, or 1/2 glucose + 1/2 fructose; coded as “S” [only sucrose], “M” [mix of sucrose and hexoses], and “H” [only hexoses], respectively). Values of total sugar concentration used in this study (15% or 50% w/v) resemble those commonly found in nectar from different plant species, which typically range from 15 to 40% w/v but may reach > 50% (e.g., under warm conditions and low relative humidity) [37–39]. Moreover, the sugar composition and ratios considered in this study (only sucrose, 1/3 sucrose + 1/3 glucose + 1/3 fructose, or 1/2 glucose + 1/2 fructose) correspond to the sucrose dominant nectar, balanced nectar, and fructose-glucose dominant nectar categories (S, SFG, and FG types, respectively) established by Percival [40] for natural nectars. Additionally, the amino acid concentration of nectar has been observed in ranges from 0.3 to 12.5 µmol/mL in naturally pollinated plants, depending on the pollinator type [41, 42]. Assuming an average molecular weight of 136.9 g/mol and an average nitrogen content of 14.7% for proteinogenic amino acids, those previously reported amino acid values correspond to 6 ppm and 251.6 ppm of amino nitrogen, respectively (but note that floral nectar may contain non-proteinogenic amino acids and other nitrogen sources). Therefore, the nitrogen content of the artificial nectars used in this study (3.48/1.67 ppm and 348/167 ppm of total nitrogen/amino nitrogen in the “n” and “N” treatments, respectively) is also similar to the content found in natural nectars. A three-letter code was used to name each artificial nectar depending on these three basic properties (Table 1).

**Table 1:** Characteristics of the artificial nectars used in this study for testing the growth performance of *Acinetobacter* and *Rosenbergiella* isolates

Artificial nectar code	Sugar level^a^	Nitrogen level^b^	Sugar composition^c^
snS	Low (15%)	Low (3.48/1.67)	S
sNS	Low (15%)	High (348/167)	S
SnS	High (50%)	Low (3.48/1.67)	S
SNS	High (50%)	High (348/167)	S
snM	Low (15%)	Low (3.48/1.67)	1/3 S, 1/3 G, 1/3 F
sNM	Low (15%)	High (348/167)	1/3 S, 1/3 G, 1/3 F
SnM	High (50%)	Low (3.48/1.67)	1/3 S, 1/3 G, 1/3 F
SNM	High (50%)	High (348/167)	1/3 S, 1/3 G, 1/3 F
snH	Low (15%)	Low (3.48/1.67)	1/2 G, 1/2 F
sNH	Low (15%)	High (348/167)	1/2 G, 1/2 F
SnH	High (50%)	Low (3.48/1.67)	1/2 G, 1/2 F
SNH	High (50%)	High (348/167)	1/2 G, 1/2 F

^a^Total percentage of sugars (w/v)

^b^Concentration of total nitrogen/amino nitrogen (all values are in ppm)

^c^S: sucrose; G: glucose; F: fructose

Artificial nectars were prepared as indicated in the Supplementary Methods and then added to different rows of sterile 96-well plates (180 μL per well) (BRAND GmbH + Co KG, Wertheim, Germany). A positive control (1/10 × tryptic soy broth, TSB; Merck Life Science) and a negative control (filter-sterilized distilled water) were included in all plates, and the order of the artificial nectars and controls in the rows of the plates (six for artificial nectars + two for the controls) was randomized in each replicate of the assay. Assay plates were kept in refrigeration (4 °C) overnight and then left at room temperature for 20 min until inoculation with bacterial cells. Eleven columns of the assay plates were then inoculated with 20 μL per well of a different cell suspension (*c.*10^7^ colony forming units per mL) in saline solution prepared and starved as indicated in the Supplementary Methods (i.e., eleven isolates were tested per plate), whereas the wells of the twelfth column were inoculated with 20 μL of sterile saline solution to serve as microbe-free controls. The order of isolates and the microbe-free control in the columns of the plates was randomized in all assay plates. All assays (growth of each *Acinetobacter* or *Rosenbergiella* isolate in each artificial nectar) were repeated at least three times, and some randomly chosen isolates were inoculated in two different columns of the same plate to test for intraplate reproducibility of the assays. Inoculated plates were covered with a breathable membrane (Breath-Easy; Diversified Biotech, Boston, MA, USA) and incubated with no agitation for 7 days at 25 °C. Optical density (OD) values at a wavelength of 600 nm were determined for each isolate and test condition by putting the assay plates into a benchtop spectrophotometer (Multiskan GO; Thermo Fisher, Merelbeke, Belgium) just after inoculation (day 0) and after 3 and 7 days of incubation; a brief shaking (30 s) was applied to each assay plate just before the OD determinations. Normalized OD readings were calculated by subtracting the OD values of the microbe-free control wells from the OD values of the test wells.

### Phylogenetic Reconstruction

Maximum likelihood (ML) phylogenetic trees were generated for all *Acinetobacter* and *Rosenbergiella* isolates and sequence types (or genotypes; STs), defined as sequences (*rpoB* for *Acinetobacter* spp. and concatenation of *atpD*, *gyrB*, and *rpoB* sequences for *Rosenbergiella* spp.) differing in at least one nucleotide, as indicated in Supplementary Methods. Briefly, nucleotide sequences obtained for the studied isolates and some reference strains (e.g., *Acinetobacter calcoaceticus* NIPH 2245^ T^ and *Phaseolibacter flectens* ATCC 12775^ T^, used as outgroups in the trees built for *Acinetobacter* and *Rosenbergiella*, respectively) were included in multiple alignments generated by MUSCLE [43]. ML trees of isolates and STs were built for the *rpoB* gene sequences of *Acinetobacter* (861 bp) and a concatenation of *atpD* + *gyrB* + *rpoB* sequences of *Rosenbergiella* (1863 bp) using PhyML v.3.0 [44] with smart model selection based on the Akaike information criterion (AIC) [45] (see details in Figs. S1 and S2), and the resulting trees were visualized and edited with the Molecular Evolutionary Genetics Analysis v.11 (MEGA11) software [46].

Additionally, to assess the phylogenetic conservatism of measured traits at the species level (see “Data Analysis”), a ML genome-based tree was generated for the two *Acinetobacter* and the six *Rosenbergiella* species included in this study using the up-to-date bacterial core gene set (UBCG2) pipeline [47] (Supplementary Methods).

### Data Analysis

Unless otherwise indicated, all data analyses described in the following sections were performed using R version 4.1.0 [48] run on RStudio v.1.4.1717 [49]. Required R libraries are indicated below.

#### Exploratory Data Analysis and Data Normalization

The growth performance (GP) of each isolate in each artificial nectar after 3 and 7 days of incubation was evaluated by subtracting to the increase in the normalized OD value (see above) obtained in such condition (ΔOD) the increase in the normalized OD value obtained in the negative control that did not contain any sugar or nitrogen source ((ΔOD_ctrl_) which accounted for possible growth due to, for example, nutrient reserves remaining after the starvation step [32]); i.e., GP = ΔOD − ΔOD_ctrl_ = (OD_f_ − OD_0_) − (OD_f-ctrl_ − OD_0-ctrl_), where OD_0_ and OD_f_ are the OD values on day 0 and after incubation (3 or 7 days), respectively, and OD_0-ctrl_ and OD_f-ctrl_ are the equivalent values obtained for the negative control.

Intra-plate reproducibility of the assay testing for growth in artificial nectars (i.e., agreement of the GP values obtained for replicates ran in the same plate) was evaluated by calculating Lin’s concordance correlation coefficient (CCC) for agreement on continuous measures, which quantifies the agreement between two measures of the same variable and ranges from − 1 (strong discordance) to 1 (perfect agreement) [50, 51]. For each combination of artificial nectar and incubation time, Lin’s CCC was calculated using the epi.ccc() function of the R library “epiR” v. 2.0.33 [52]. Scatter plots of the replicate data and the corresponding linear regression line were generated using “ggplot2” v.3.3.5 [53].

All trait values were further processed by calculating the average of the GP values obtained for the three replicates of each isolate and test condition (GP_avg_), and the distributions of GP_avg_ values obtained in each test condition for *Acinetobacter vs. Rosenbergiella* and the different species tested were visualized by violin plots using the R library “vioplot” v.0.3.7 [54]. To allow a fairer comparison of growth data across genera and species, GP_avg_ values were converted into *Z*-scores (i.e., GP_avg_ of a particular isolate minus average GP_avg_ of all 88 isolates (43 *Acinetobacter* + 45 *Rosenbergiella* isolates) for the same test, divided by the corresponding standard deviation) using Microsoft Excel 2016 (Redmond, WA, USA). For a given test condition (artificial nectar and incubation time), isolates with positive *Z*-scores had a growth performance above the mean of the studied set of isolates, whereas those with negative *Z*-scores had a growth performance below the mean. Finally, the mean *Z*-score of the isolates belonging to each ST and species of *Acinetobacter* and *Rosenbergiella* was calculated.

#### Nonparametric Factorial Analysis of Variance

The effect of different factors — namely, taxonomic affiliation (at the genus or species level), incubation time (3 or 7 days), and the type of artificial nectar (Table 1) — and their interactions on the growth results of all the isolates in the different types of artificial nectar (expressed as a vector of *Z*-scores) was analyzed by repeated-measures factorial analysis of variance (ANOVA) of aligned rank transformed (ART) data, as implemented in the R library “ARTool” [55, 56]. ART-based ANOVA is a non-parametric statistical test that relies on a preprocessing step that “aligns” the data for each possible main effect or interaction (i.e., effects are estimated as marginal means and then stripped from the response variable so that all effects but one are removed) before assigning averaged ranks, after which conventional ANOVA procedures are used [55, 56]. *P* values < 0.05 were considered significant.

#### Phylogenetically Informed Analyses

Further analysis of growth performance data was achieved by applying different methods which account for the non-independence of data due to shared ancestry. Such phylogenetically informed methods were applied at the isolate, genotype (ST), and species level to evaluate the magnitude of phylogenetic correlations in studied traits. The inputs of the analyses were (i) an R data frame with the *Z*-scores (or average *Z*-scores) obtained for *Acinetobacter* and *Rosenbergiella* isolates, STs, or species, and (ii) the ML phylogenetic trees built from housekeeping gene sequences or genome assemblies, and converted into ultrametric trees (i.e., trees where all tips are at equal distance from the root) by the force.ultrametric() function of “phytools” v.0.7–80 [57]. When necessary, phylogenetically independent contrasts (PICs) [58] were calculated by applying the pic() function of the R library “ape” v.5.5 [59] to the *Z*-scores obtained for each trait (growth in different artificial nectars after 3 and 7 days of incubation). Holm’s correction was applied to *P* values whenever multiple comparisons were made, and corrected *P* values < 0.05 were considered significant.

Pairwise correlations between PICs obtained for different traits for *Acinetobacter* and *Rosenbergiella* isolates were assessed by the non-parametric Spearman rank test, as implemented in the R library “Hmisc” v.4.5–0 [60]. Correlation matrices were visualized using the R library “corrplot” v.0.90 [61].

Visualization of the trait data obtained for obtained for *Acinetobacter* and *Rosenbergiella* isolates, STs, and species on the corresponding ML trees was achieved by generating phylogenetic heatmaps using the phylo.heatmap() function of “phytools.” In addition, we used the fitContinuous() function of the R library “geiger” v.2.0.7 [62] to determine which model of trait evolution provided the best fit to the phenotypic data. Nine different models were tested (Brownian motion, Ornstein–Uhlenbeck, early-burst, trend, Pagel’s *λ*, Pagel’s *κ*, Pagel’s *δ*, drift, and white noise model; see Supplementary Methods), and their relative likelihood was assessed by calculating their AIC values and Akaike weights [63]. When no evolutionary model yielded an Akaike weight ≥ 0.5, it was concluded that none of them fit the data substantially better than the others [32, 36, 64, 65].

Finally, the statistical dependence among the *Z*-scores obtained for isolates, STs, and species due to their phylogenetic relationships was evaluated by calculating four different phylogenetic signal metrics commonly used in comparative biology, namely, Blomberg’s *K*, Pagel’s *λ*, Moran’s *I*, and Abouheif’s *C*_mean_ [36, 65–73]. *K* and *λ* assume a Brownian motion model of trait evolution (i.e., random walk with constant trait variance over time [58]), and the closer their values are to zero, the more phylogenetically independent a trait is, while values of 1 for these metrics correspond to the Brownian motion expectation, and values > 1 mean that close relatives are more similar than expected under Brownian motion [69, 70]. Therefore, *K* and *λ* can be used to assess the strength (or “effect size”) of phylogenetic structuring [67, 72]. In contrast, *I* and *C*_mean_ are autocorrelation indices that depend on the structure and size of the phylogeny, but which are not based on any evolutionary model and are unable to provide information on the strength of the phylogenetic signal [67, 72]. Therefore, the values of *I* and *C*_mean_ cannot be quantitatively compared [72]. Computation of all these metrics was performed using the multiPhylosignal() function of R library “picante” v.1.8.2 [74] for calculation of *K*, the phylosig() function of “phytools” for *λ*, and the abouheif.moran() function of “adephylo” v.1.1–11 [75] for *I* and *C*_mean_ (method = “Abouheif” and “oriAbouheif” for computation of phylogenetic proximity between the tips of trees, respectively). Statistical significance was tested in all cases by randomization with 1000 repetitions.

## Results

### Identification of Sequence Types and Phylogenetic Reconstruction

Analysis of *rpoB* sequences identified a total of 18 STs (ST01A to ST18A) among the *Acinetobacter* isolates included in this study, whereas 24 STs (ST01R to ST24R) were identified for the concatenation of *atpD* + *gyrB* + *rpoB* sequences among the *Rosenbergiella* isolates (Tables S1 and S2). STs represented between one and ten conspecific isolates of nectar or bee origin, but never from both habitats. However, two *Acinetobacter* and five *Rosenbergiella* STs included isolates from different plant species, and one *Rosenbergiella* ST grouped two isolates retrieved from honeybees with one obtained from a bumblebee (Tables S1 and S2).

In general, isolates or STs belonging to the same species, as determined by phylogenetic proximity to the corresponding type strain or a reference strain, formed well-supported clades (> 90% bootstrap) in the ML trees built for *Acinetobacter* or *Rosenbergiella* (Figs. S1 and S2). However, the combined analysis of *atpD*, *gyrB*, and *rpoB* sequences did not provide enough resolution for differentiating between the single isolate of “*R. metrosideri*” (JB07) and *R. epipactidis* isolates (Fig. S2). Furthermore, two major subclades were detected within *R. nectaris*, one of which grouped three out of the four *Rosenbergiella* isolates of bee origin analyzed in the present study (namely, B1A, B4A, and B5A) and was phylogenetically close to the fourth one (B3A) (Fig. S2). Nevertheless, the branching of such *R. nectarea* subclades was only moderately supported (77.5% bootstrap). Examination of the ML genome tree generated from the UBCG2 set confirmed the robustness of the clades grouping the type strains of the two *Acinetobacter* species and the six *Rosenbergiella* species analyzed in this study, as well as the phylogenetic relatedness between *R. epipactidis* and *‘R. metrosideri’* (Fig. S3).

### Growth in Artificial Nectars

All *Acinetobacter* and *Rosenbergiella* isolates showed very little or null growth in the nutrient-free negative control (ΔOD_ctrl_ values after 7 days of incubation were in the range of 0.000–0.045 (mean ± S.D. = 0.001 ± 0.004) for *Acinetobacter* isolates and of 0.000–0.017 (mean ± S.D. = 0.001 ± 0.002) for *Rosenbergiella* isolates). Scarce or null growth was also observed in eight of the twelve artificial nectars studied, including most of the nectars with a high sugar content and all the nectars containing the lowest nitrogen content (ΔOD_avg_ values after 7 days of incubation [ΔOD_avg7_] were < 0.05 in the following artificial nectars: snS, SnS, snM, SnM, SNM, snH, SnH, and SNH; Tables 1 and S3). Therefore, only the growth data obtained for the following four artificial nectars was considered in subsequent analyses: sNS (low sugar/high nitrogen/only sucrose; ΔOD_avg7_ = 0.005–0.489), sNM (low sugar/high nitrogen/mixture of sucrose, glucose, and fructose; ΔOD_avg7_ = 0.067–0.401), sNH (low sugar/high nitrogen/only hexoses; ΔOD_avg7_ = 0.049–0.393), and SNS (high sugar/high nitrogen/only sucrose; ΔOD_avg7_ =  − 0.009–0.071) (Table S3). Intra-plate reproducibility of the assays testing for growth in these four artificial nectars on day 3 and 7 was generally good to excellent, as the CCC 95% confidence intervals included in all cases values > 0.8 (Table S4) and the regression lines between repeated measures were close to the line of perfect concordance (i.e., *y* = *x*; Fig. S4).

**Fig. 1:**
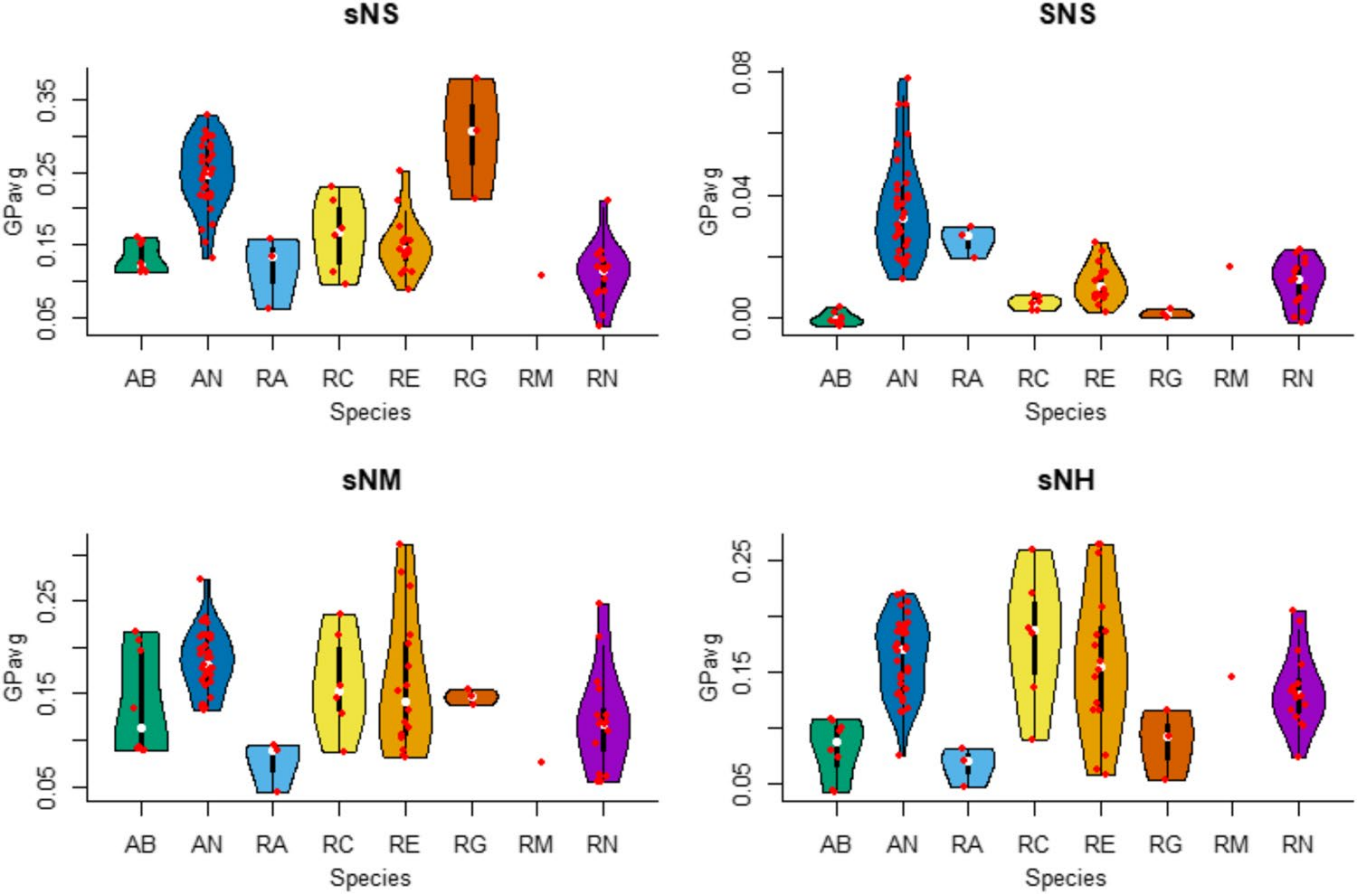
Violin plots showing the growth results, expressed as average growth performance (GP_avg_), in different artificial nectars of the *Acinetobacter* and *Rosenbergiella* species tested in this study. Note that, in general, the GP_avg_ values obtained for most species in each artificial nectar were broadly distributed around the median value (white dots contained within the thick black bars representing the interquartile range [IQR] that are shown inside the violin plots). Outlier values (red dots located beyond the boundaries of the thin black lines representing 1.5 × IQR) are evident in some plots. Abbreviations: AB, *Acinetobacter boissieri* (*n* = 8); AN, *Acinetobacter nectaris* (*n* = 35); RA, *Rosenbergiella australiborealis* (*n* = 3); RC, *Rosenbergiella collisarenosi* (*n* = 6); RE, *Rosenbergiella epipactidis* (*n* = 16); RG, ‘*Rosenbergiella gaditana*’ (*n* = 3); RM, ‘*Rosenbergiella metrosideri*’ (*n* = 1); RN, *Rosenbergiella nectarea* (*n* = 16). Artificial nectar codes are as in Table 1

In general, the average growth performance (i.e., GP_avg_) values obtained in the different artificial nectars for the isolates of each *Acinetobacter* and *Rosenbergiella* species were broadly distributed and showed several outliers (Fig. 1). ART-based ANOVA revealed that the growth of isolates in artificial nectars significantly depended on their taxonomic affiliation (at the genus and species level) and the two-way interactions between the taxonomic affiliation and the type of nectar, and between the taxonomic affiliation and the incubation time, whereas the type of nectar and incubation time had by themselves a lower and non-significant influence on growth differences (Table 2).

**Table 2:** Results obtained in the non-parametric factorial analysis of variance (ANOVA) of the growth performance of *Acinetobacter* and *Rosenbergiella* isolates in artificial nectars using aligned rank transformed data

Factors	Species-level analysis^a^				Genus-level analysis^a^			
	df	df res	*F*	*P*	df	df res	*F*	*P*
Artificial nectar (AN)^**b**^	3	560	0.38	0.768	3	602	0.50	0.685
Incubation time (IT)^c^	1	560	< 0.01	0.954	1	602	0.16	0.688
Taxonomic affiliation (TA)^d^	7	80	26.78	< 0.001*	1	86	52.43	< 0.001*
AN × IT	3	560	0.22	0.884	3	602	0.31	0.817
AN × TA	21	560	11.94	< 0.001*	3	602	13.82	< 0.001*
IT × TA	7	560	6.93	< 0.001*	1	602	22.54	< 0.001*
AN × IT × TA	21	560	1.23	0.223	3	602	0.84	0.474

^a^df: degrees of freedom; df res: degrees of freedom of residuals, *F*: *F* statistics; *P*: *P* values (statistically significant values are denoted by an asterisk)

^b^Artificial nectar types: low sugar/high nitrogen/only sucrose (sNS); low sugar/high nitrogen/mixture of sucrose, glucose, and fructose (sNM); low sugar/high nitrogen/only hexoses (sNH); and high sugar/high nitrogen/only sucrose (SNS) (see details in Table 1)

^c^Incubation time of assay plates (3 days *vs.* 7 days)

^d^Taxonomic affiliation of studied isolates at the species level (*A. boissieri* [*n* = 8], *A. nectaris* [*n* = 35], *R. australiborealis* [*n* = 3], *R. collisarenosi* [*n* = 6], *R. epipactidis* [*n* = 16], *‘R. gaditana’* [*n* = 3], *‘R. metrosideri’* [*n* = 1], or *R. nectarea* [*n* = 16]; species names pending of validation are indicated between quotation marks) or genus level (*Acinetobacter* [*n* = 43] *vs. Rosenbergiella* [*n* = 45])

### Phylogenetic-Based Analysis of Trait Variation

Correlation analysis of the PICs obtained from *Z*-scores data revealed 11 and 8 significant correlations between the assimilation assays for *Acinetobacter* and *Rosenbergiella*, respectively (39.3% and 28.6%, respectively, of the total number of pairwise comparisons, *n* = 28) (Fig. S5). All significant correlations obtained for *Acinetobacter* and *Rosenbergiella* were positive (Spearman’s *ρ* = 0.513–0.817 and 0.695–0.868, respectively). Moreover, the PICs obtained for growth in a same artificial nectar after 3 and 7 days of incubation were highly correlated (Fig. S5), so subsequent phylogenetic-based analyses were only performed using the results obtained on day 3.

**Fig. 2:**
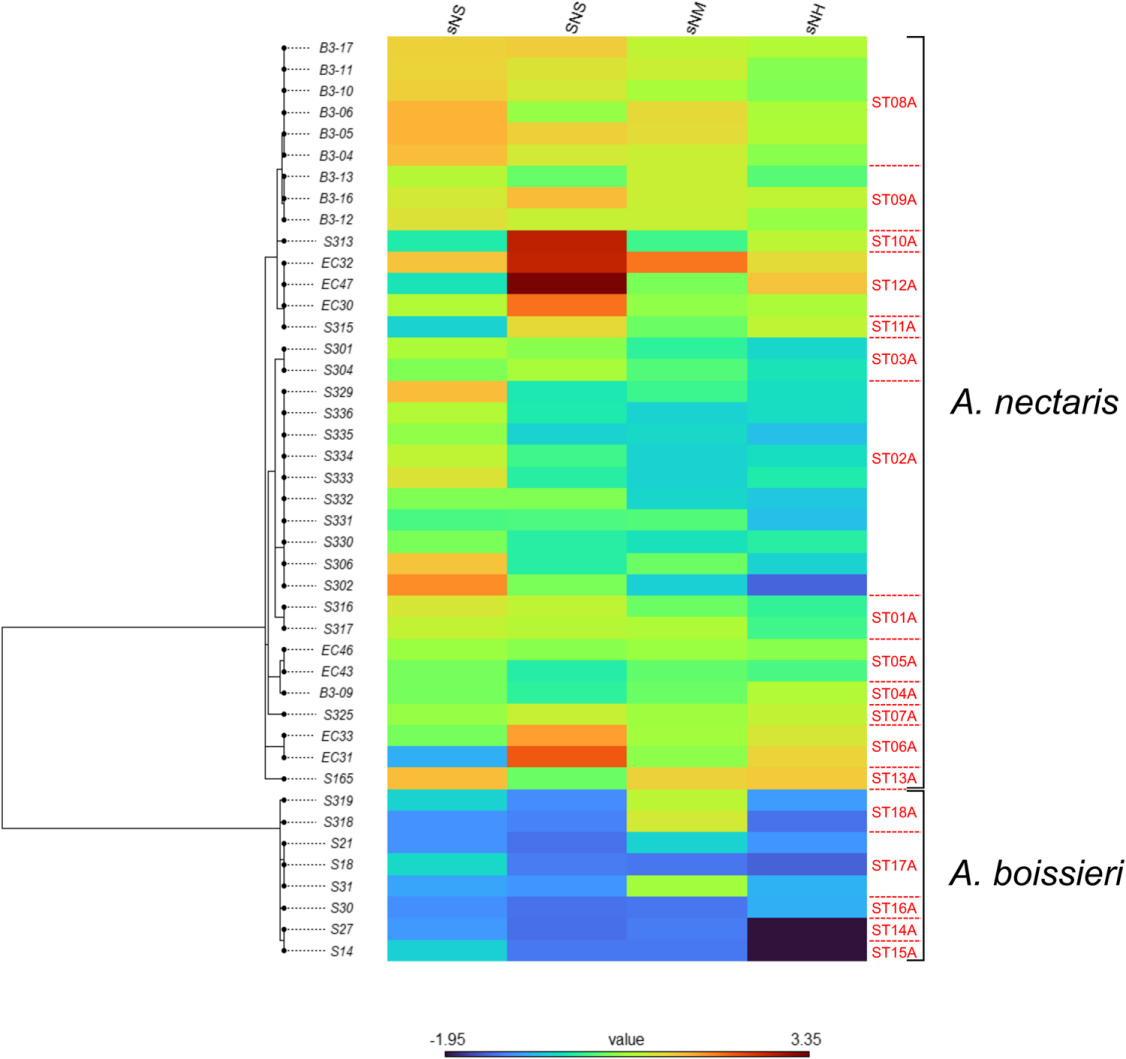
Phylogenetic heatmap of the trait values obtained for the different *Acinetobacter* isolates (shown in rows) in the growth assays performed in this study. Artificial nectar codes (shown in columns) are as in Table 1. The phylogenetic tree shown on the left corresponds to the maximum likelihood (ML) tree depicted in Fig. S1. The sequence type (ST; shown in red characters) and species affiliation of isolates are indicated at the right side of the heatmap (see also Table S1). Note that some clade dependence of the trait values (growth in the four artificial nectars under analysis) is observed, with *A. boissieri* isolates displaying in most cases lower trait values than *A. nectaris*

Mapping of the trait data to the ML trees generated for *Acinetobacter* suggested some clade dependence of the trait values. In particular, *A. boissieri* isolates and STs displayed lower growth values in most test conditions than *A. nectaris* (see the phylogenetic heatmaps in Figs. 2 and S6). Furthermore, some intraspecies differentiation was found within *A. nectaris* and *A. boissieri* at the isolate and ST level (Figs. 2 and S6). Evolutionary model fitting of studied traits indicated that Pagel’s *κ* and *λ* models were the most supported for *Acinetobacter* isolates and STs (each model fit the *Z*-scores obtained for two artificial nectars) (Table S5). Phylogenetic signal analysis of the *Z*-scores obtained for *Acinetobacter* isolates and STs yielded significant results for all artificial nectars when tested by Moran’s and Abouheif’s methods, and by Pagel’s method (100% of significant tests, in all cases; Table 3). Blomberg’s *K* values were low (1.12·10^−6^–5.67 × 10^−6^) but significant for all traits when analyzed for *Acinetobacter* isolates, and non-significant for all traits except growth in the sNH nectar after 3 days of incubation (*K* = 1.35 × 10^−4^) when analyzed for STs (Table 3).

**Table 3:** Phylogenetic signal of the growth of *Acinetobacter* and *Rosenbergiella* isolates, sequence types (STs), and species in different artificial nectars

Phylogenetic tree (*N*)^a^	Phylogenetic signal metric^b^	Artificial nectars^c,d^			
		sNS	SNS	sNM	sNH
*Acinetobacter*					
Isolates (43)	*K*	2.23 × 10^−6^ (0.004*)	4.52 × 10^−6^ (0.004*)	1.12 × 10^−6^ (0.004*)	5.67 × 10^−6^ (0.004*)
	λ	0.901 (< 0.001*)	0.980 (< 0.001*)	0.870 (0.001*)	0.983 (< 0.001*)
	*I*	0.573 (0.004*)	0.760 (0.004*)	0.499 (0.004*)	0.723 (0.004*)
	*C*_mean_	0.595 (0.004*)	0.769 (0.004*)	0.520 (0.004*)	0.742 (0.004*)
STs (18)	*K*	3.21 × 10^−6^ (0.541)	5.38 × 10^−6^ (0.541)	8.76 × 10^−6^ (0.527)	1.35 × 10^−4^ (0.024*)
	λ	0.642 (0.004*)	0.945 (< 0.001*)	0.433 (0.033*)	0.998 (< 0.001*)
	*I*	0.427 (0.004*)	0.594 (0.004*)	0.501 (0.004*)	0.654 (0.004*)
	*C*_mean_	0.491 (0.004*)	0.613 (0.004*)	0.557 (0.004*)	0.691 (0.004*)
*Rosenbergiella*					
Isolates (45)	*K*	1.05 × 10^−6^ (0.026*)	1 × 10^−6^ (0.004*)	2 × 10^−6^ (0.004*)	1.84 × 10^−6^ (0.004*)
	λ	0.834 (< 0.001*)	0.797 (0.002*)	0.982 (0.025*)	0.911 (0.022*)
	*I*	0.439 (0.004*)	0.446 (0.004*)	0.406 (0.004*)	0.452 (0.004*)
	*C*_mean_	0.480 (0.004*)	0.460 (0.004*)	0.417 (0.004*)	0.467 (0.004*)
STs (24)	*K*	0.042 (0.086)	0.105 (0.004*)	0.036 (0.086)	0.041 (0.075)
	Λ	0.564 (1)	0.973 (0.006*)	6.62 × 10^−5^ (1)	6.62 × 10^−5^ (1)
	*I*	− 0.078 (0.572)	0.344 (0.020*)	0.033 (0.5)	0.187 (0.152)
	*C*_mean_	0.064 (0.551)	0.394 (0.020*)	0.061 (0.551)	0.224 (0.120)
Species (8)	*K*	0.099 (0.888)	0.154 (0.444)	0.044 (0.888)	0.131 (0.483)
	λ	6.68 × 10^−5^ (1)	6.68 × 10^−5^ (1)	6.68 × 10^−5^ (1)	6.68 × 10^−5^ (1)
	*I*	− 0.257 (1)	− 0.414 (1)	− 0.278 (1)	− 0.328 (1)
	*C*_mean_	− 0.103 (1)	− 0.099 (1)	− 0.099 (1)	− 0.084 (1)

^a^Phylogenetic tree used for computation of phylogenetic signal indices (see details in the main text). *N*: number of isolates, STs, or genomes available for analysis

^b^*K*: Bloomberg’s *K*; *λ*: Pagel’s *λ*; *I*: Moran’s *I*; *C*_mean_: Abouheif’s *C*_mean_

^c^Artificial nectar codes are as in Table 1

^d^Phylogenetic signal metric estimates and Holm-corrected *P*-values. Significant *P* values (< 0.05) are denoted by an asterisk

**Fig. 3:**
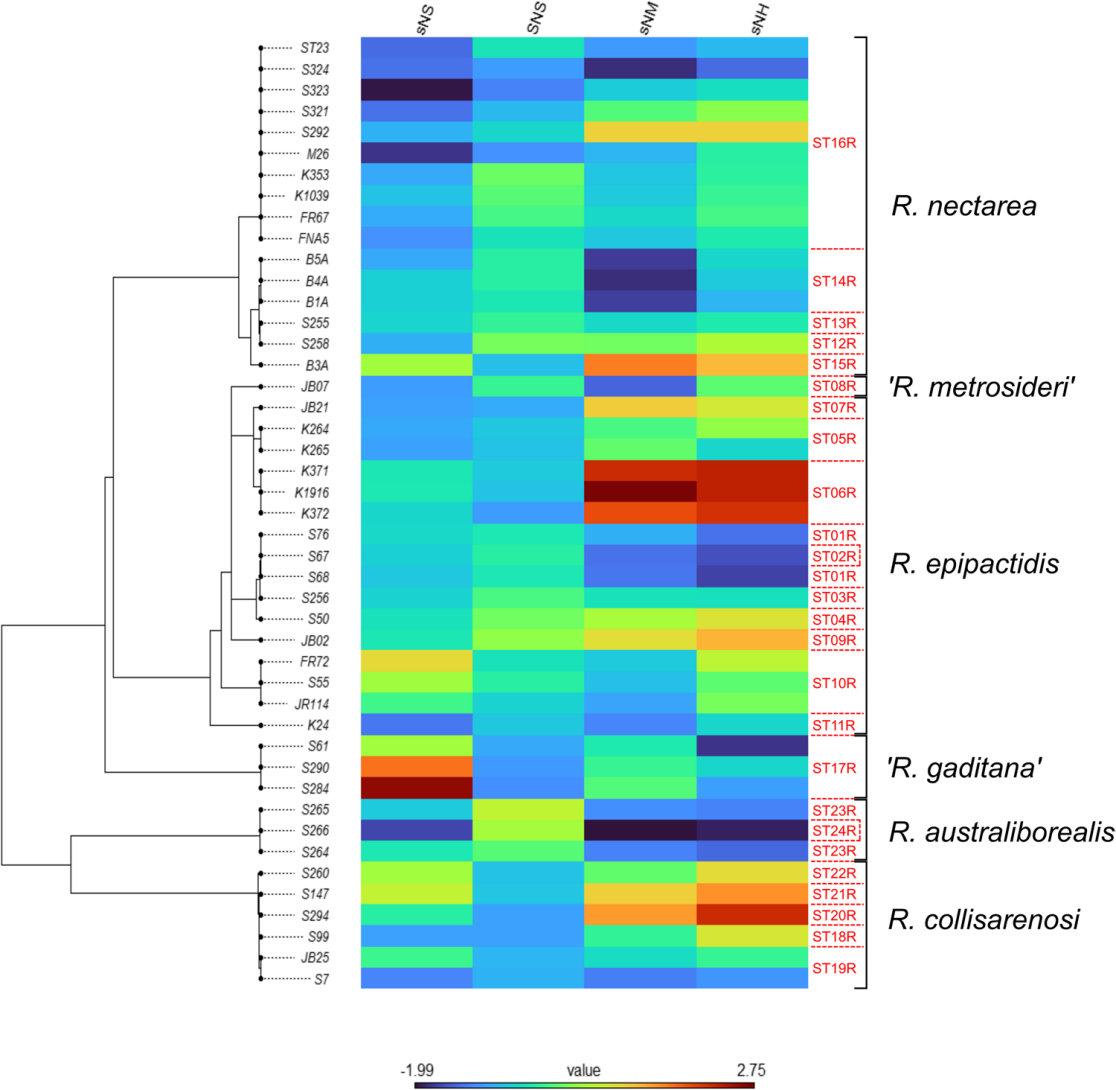
Phylogenetic heatmap of the trait values obtained for the different *Rosenbergiella* isolates (shown in rows) in the growth assays performed in this study. Artificial nectar codes (shown in columns) are as in Table 1. The phylogenetic tree shown on the left corresponds to the maximum likelihood (ML) tree depicted in Fig. S2. The sequence type (ST; shown in red characters) and species affiliation of isolates is indicated at the right side of the heatmap (see also Table S2). Species names pending of validation are indicated between quotation marks. Note that trait differentiation between clades is less evident than for *Acinetobacter* isolates (see Fig. 2), as closely related isolates displayed in some cases divergent trait values

Trait differentiation between clades at the isolate and ST level was less evident in the phylogenetic heatmaps generated for *Rosenbergiella* ML (Figs. 3 and S7). Pagel’s *κ* was the most supported evolutionary model for *Rosenbergiella* isolates (*Z*-scores obtained in 3 out of 4 artificial nectars), followed by Pagel’s *λ* model (*Z*-scores obtained in the sNS nectar). In contrast, the *Z*-scores obtained for *Rosenbergiella* STs fit in most cases a white noise model of evolution, whereas Pagel’s *κ* was only supported for growth in the SNS nectar (Table S5). Significant results were obtained for all artificial nectars and all phylogenetic signal metrics at the isolate level (Table 3). Blomberg’s *K* values were low (< 2 × 10^−6^) in all cases, whereas high *λ* values were obtained for growth in the sNM and the sNH nectars (*λ* > 0.91). ST-based analysis yielded significant results for all phylogenetic signal metrics for growth in the SNS nectar, but not in the other nectars containing a lower sugar concentration (sNS, sNM, and sNH; Table 3).

Mapping of the trait data to the ML tree generated from genome sequences showed no clear pattern of trait variation across *Rosenbergiella* species but confirmed the phenotypic differences between *Acinetobacter* species (*A. nectaris* displayed higher average growth values in all test conditions than *A. boissieri*; Fig. S8). The *Z*-scores obtained for *Acinetobacter* and *Rosenbergiella* species usually best fit a white noise model, whereas no evolutionary model of trait evolution performed substantially better that the others in the analysis of the *Z*-scores obtained for the SNS nectar (Table S5). No phylogenetic signal metric yielded significant results for any artificial nectar in the species-level analysis (Table 3).

## Discussion

The high osmotic pressure and highly unbalanced carbon to nitrogen ratio often found in floral nectar are major hurdles to microbial life that determine the assembly of microbial communities in this microhabitat [9–12, 14, 17, 32]. However, there is still limited information on how different microorganisms respond to different types of nectar. The phylogenetic determination of growth variation in floral nectar was addressed in the present study by analyzing the growth performance of a diverse collection of *Acinetobacter* and *Rosenbergiella* isolates in artificial nectars differing in three basic chemical properties, namely, overall sugar content, overall nitrogen content, and sugar composition.

A first important result of this study was that all the *Acinetobacter* and *Rosenbergiella* isolates tested did not grow or only displayed very scarce growth in the artificial nectars containing the lowest amount of nitrogen (3.48 ppm), suggesting that these bacteria may preferentially grow in nitrogen-rich nectars. Unfortunately, to our knowledge, no published report on bacterial presence in floral nectar includes information about the nitrogen content of nectar samples, so the hypothetical preference of *Acinetobacter* spp. and *Rosenbergiella* spp. for nitrogen-rich nectars cannot be confirmed or refuted with field data. Additionally, it should be noted that even though the nectar-inhabiting representatives of these bacterial genera seem unable to grow in low-nitrogen media, they might be adapted to survive nitrogen limitation in a quiescent state and resume growth upon arrival of nitrogen inputs from external sources (e.g., pollen grains that fall into nectar or the frass of insects that visit flowers), the metabolic by-products of other nectar microbes such as yeasts, and/or cell debris [17, 32, 76]. For example, it has been recently demonstrated that the *Acinetobacter* species typically found in floral nectar can induce pollen germination and bursting, and protein release into solution, which might benefit bacterial growth [76].

Furthermore, our results confirmed that the high osmotic pressure resulting from elevated sugar concentrations is an efficient filter of microbial growth in floral nectar [10], as this factor hindered the growth of most isolates that were analyzed. In fact, negligible growth of all *Acinetobacter* and *Rosenbergiella* isolates was obtained in the SNM and SNH nectars, which contained the highest sugar level (50% w/v), a high nitrogen content (348 ppm in total), and either a mixture of sucrose and hexoses (SNM) or only hexoses (SNH). Nevertheless, some isolates grew in the SNS nectar that had the same overall sugar and nitrogen content as SNM and SNH but included sucrose as the only sugar source. Nectar sugar concentration varies significantly in natural plant populations, both within and between species, and may be affected by microclimatic conditions [77, 78]. Most plant species have nectar sugar concentrations ranging from 15 to 40% w/v, whereas concentrations over 50% are only frequent under warm conditions and low relative humidity [37–39]. Results of the present study thus prompt the testable prediction that densities of *Acinetobacter* and *Rosenbergiella* in nectar, and perhaps other nectar bacteria too (see Pusey [79]), should decline in seasons, habitats, or regions characterized by consistently warm and dry conditions. However, Von Arx et al. [80] observed that the abundance of nectar bacteria in two night-blooming plants of the Sonoran Desert reached in some cases 10^6^ CFU/mL, which is within the range of values reported in studies performed in temperate regions [6, 24]. Moreover, drought often does not result in more concentrated nectar [81], and it has been observed that some bacteria such as *Fructobacillus* (phylum *Bacillota*) grow better in synthetic nectar at warmer temperatures [82]. It should also be noted that the molar concentration of sugar solutions with the same percentage of sugar (% w/v) differs depending on the monosaccharide content: the higher the monosaccharide content, the higher the molarity. Since water activity (*a*_*w*_) depends on molarity, the monosaccharide solutions will have a lower *a*_*w*_, which may explain why amongst the high sugar nectars, only the one with only sucrose (i.e., SNS) was able to support some growth. To our knowledge, most previous osmotolerance tests carried out on the *Acinetobacter* and *Rosenbergiella* species analyzed in this study have incorporated sucrose as the only sugar source [27, 30, 31, 33, 35]. Specific solute effects of sucrose, glucose, and fructose have been reported for other bacterial species and could lead to differential responses to osmotic stress [83]. Additionally, the lack of growth in the SNM and SNH nectars might not only be due to the high osmotic pressure resulting from elevated monosaccharide concentrations, but also to other factors such as the differences in viscosity and/or dissociation constants (which result in pH changes) of the different sugar types making up the solution mixture [84].

A common expectation in trait biology in scenarios where evolution mainly proceeds by vertical gene inheritance is that closely related organisms resemble each other more than compared to distant relatives [65, 69]. However, events such as gene loss, horizontal gene transfer — which is particularly common among the *Pseudomonadota* [85]—, and convergent evolution may reduce the phylogenetic signal of functional traits [86]. In this regard, the ANOVA results obtained in this study indicated that growth variation in artificial nectars mostly depended on the genus and species identity of tested isolates, whereas the nectar type and the incubation time of test plates explained a limited amount of the total variation in the data, mostly in interaction with the taxonomy of isolates. Within genus *Acinetobacter*, phenotypic differentiation between *A. nectaris* and *A. boissieri* was evident when the trait data obtained in the present study was mapped onto the ML trees built from *rpoB* sequences and the UBCG2 set. Furthermore, significant phylogenetic signal was detected for growth in the sNS, sNM, sNH, and SNS nectars by most methods of analysis, thus suggesting that the distribution of trait values among *Acinetobacter* isolates and STs was not random but significantly correlated with phylogeny. Nevertheless, evolutionary model fitting and the low Blomberg’s *K* values obtained for all traits under study for *Acinetobacter* isolates and STs suggest that the evolution of such traits departed from a pure Brownian motion. The evolutionary models most supported for traits with significant phylogenetic signal were Pagel’s *κ* and *λ*, both of which are transformations of the Brownian motion model that raise all branch lengths in the phylogenetic tree to a power *κ* or multiply internal branches by a factor *λ*, respectively (but note that *κ* = 1 and *λ* = 1 correspond to pure Brownian motion) [73]. Similar phenotypic differentiation between *A. nectaris* and *A. boissieri* and departure from a pure Brownian motion model has been recently reported for assimilation of diverse nitrogen sources [32], but the physiological mechanisms responsible for the growth differences between these and other recently described nectar-inhabiting species of *Acinetobacter* (*A. pollinis*, which seems to be the closest relative to *A. nectaris*, and *A. baretiae* and *A. rathckeae*, which belong to the same clade as *A. boissieri* [27]) remain to be elucidated.

Significant phylogenetic signal was also obtained for growth of *Rosenbergiella* in all artificial nectars at the isolate level and for growth in the SNS nectar at the ST level, even when no clear phenotypic differentiation was found between the representatives of this genus in the phylogenetic heatmaps generated from the concatenation of housekeeping gene sequences or the UBCG2 set. In contrast, no significant phylogenetic signal was detected for any trait in the species-level analysis. Moreover, the growth data obtained for *Rosenbergiella* STs and *Acinetobacter* and *Rosenbergiella* species in the sNS, sNM, and SNH nectars best fit the white noise model of trait evolution, which assumes that trait values follow a random normal distribution and species have no significant trait covariance [65]. Altogether, these results suggest that the ability to grow in floral nectar is highly conserved between closely related isolates and genotypes of *Acinetobacter* and *Rosenbergiella*, but this pattern vanishes deeper in the phylogeny (i.e., between congeneric species or between the genera *Acinetobacter* and *Rosenbergiella*). It remains unclear how this hypothesis fits within the framework for predicting the phylogenetic conservatism of functional traits proposed by Martini et al. [87], by which the dispersion and the depth of clades that contain a given trait are correlated with its complexity, so that complex traits encoded by many genes are highly phylogenetically conserved and found in a few deep clades, whereas simpler traits (e.g., the ability to use simple nutrients) are phylogenetically dispersed and found in small clades.

Habitat differences are often linked to trait variation, as different environmental conditions can select for different phenotypes. For example, Pozo et al. [13] found that nectar-inhabiting isolates of the yeasts *Metschnikowia reukaufii* and *Metschnikowia gruessii* that originated from a same plant host (at the family and/or species level) tended to show more similar overall growth profiles in media containing different nutrient sources or growth inhibitors than did isolates from other hosts. Moreover, we recently reported that, in general, *Acinetobacter* isolates of bee origin showed higher growth than nectar isolates in media containing as the only nitrogen source L-cysteine or L-tryptophan [32]. Given the different microenvironmental conditions of floral nectar and the digestive tract of bees (e.g., sugar-dominated, nitrogen-poor chemical composition *vs.* complex chemical composition, determined by the presence of dietary and excretion waste, respectively), it may be expected that bacterial isolates from these habitats display some phenotypic differences. However, as our collection of isolates was biased towards isolates from a limited selection of plant families and bee species collected in a few countries, the habitat and geographic origin of isolates were not taken into account in our data analysis. A detailed characterization of additional isolates from different animal visitors of flowers (including different insect orders and non-insect vectors of microbes) and more diverse plant hosts (e.g., including plant species from tropical regions, which seem to be important reservoirs of nectar microbes [88]) is required for a fair habitat-based comparison of bacterial phenotypes.

Previous research has revealed that trait variation in *Metschnikowia* yeasts is to some extent determined by the total sugar concentration and the relative fructose content in the floral nectar of the host plant [12]. In the present study, we did not analyze the chemical composition of the nectar samples from which the studied isolates were obtained and, therefore, we could not determine the impact of the host’s nectar chemistry on the growth performance of *Acinetobacter* and *Rosenbergiella*. Moreover, the floral nectar of many plant species contains antimicrobial proteins and secondary compounds putatively protecting nectar from microbial invasion [12, 89–93], a factor which was not considered in the preparation of the artificial nectars used in this study. However, the chemical composition of floral nectar varies widely at the interspecies and intraspecies level (and even between flowers of a same plant or the nectaries of a same flower), and can depend on climatic conditions [15, 94, 95]. Additionally, nectar microbes can modify nectar chemistry, either by consuming sugars or other nutrients, by modifying its pH, by releasing diverse metabolites, and/or by reducing the concentration of some toxins of plant origin [93, 96, 97]. Therefore, determining if trait variation in nectar microbes is linked to nectar chemistry might be challenging. Finally, for the sake of simplification, the growth experiments carried out in this study included cells of a single isolate of *Acinetobacter* or *Rosenbergiella*. However, different genotypes and/or species of these genera frequently co-occur in floral nectar, and they can also coexist with other bacteria and yeasts [18, 26]. Therefore, our experiments did not account for possible mechanisms of microbe-microbe interaction resulting in growth facilitation or inhibition that may take place in nature [9]. In any case, to our knowledge, this is the first study demonstrating that phylogenetic factors determine the ability of nectar bacteria to grow in artificial media mimicking the conditions of osmotic pressure and nitrogen limitation found in natural nectars. As many relevant traits of nectar bacteria, such as their ability to alter nectar’s chemistry and interact with floral visitors (e.g., through the production of volatile organic compounds), depend on their osmotolerance and nitrogen scavenging ability, we consider that bacterial phylogeny should be incorporated as a variable in future studies analyzing the ecological relevance of these microorganisms.

## Conclusion

In conclusion, the results of this study demonstrate that elevated sugar concentration and low nitrogen content are major hurdles for the growth of *Acinetobacter* and *Rosenbergiella* in nectar. Furthermore, our results reveal that the ability of these microorganisms to grow in different types of nectar is highly conserved across closely related isolates and genotypes (STs), but this conservatism rapidly vanishes deeper in phylogeny. The importance of other factors, such as the habitat and geographical origin of isolates, in determining the phenotypic differentiation between isolates, STs, and species of nectar- and/or bee-associated bacteria remains to be evaluated in future studies.

## Supplementary Information

Below is the link to the electronic supplementary material.

Supplementary file1 (DOCX 32 KB)

Supplementary file2 (PDF 337 KB)

Supplementary file3 (PDF 843 KB)

## Data Availability

All relevant results are included in this paper or available as supplementary materials at the journal’s website. The sequence data used in this study is available at the GenBank/ENA/DDBJ databases under the accession numbers shown in the main text, supplementary methods, and/or Tables S1 and S2.

## Declarations

### Disclaimer

The funders had no role in the preparation of the manuscript or in the decision to publish.
