# Supplementary material for: Sugar Concentration, Nitrogen Availability, and Phylogenetic Factors Determine the Ability of Acinetobacter spp. and Rosenbergiella spp. to Grow in Floral Nectar
Source: Microb Ecol. 2022 Aug 5;86(1):377–91. doi: 10.1007/s00248-022-02088-4 (PMC10293439; doi:10.1007/s00248-022-02088-4)
Supplement: Supplementary file 1 — Supplementary file1 (DOCX 32 KB) [file 248_2022_2088_MOESM1_ESM.docx]

**SUPPLEMENTARY METHODS**

**Species-level classification and conservation of isolates**

Species-level classification of the 43 *Acinetobacter* isolates used in the present study was performed by BLASTn searches against GenBank of partial sequences obtained in previous studies for the gene encoding the β subunit of RNA polymerase (*rpoB*) [1, 2] (accession numbers are shown in Table S1). Similarly, the 45 *Rosenbergiella* isolates were classified into species by BLASTn searches of the partial sequences previously obtained for the following three housekeeping genes: *atpD*, which encodes the ATP synthase β-chain; *gyrB*, encoding the DNA gyrase subunit B; and *rpoB* [3, 4] (Table S2).

**Preparation of artificial nectars**

The 12 artificial nectars used in this study (see details in Table 1) were prepared by dissolving the corresponding amount of each sugar (sucrose, glucose, and/or fructose; Merck Life Science, Overijse, Belgium) and peptone (peptone from casein, pancreatic digest; total nitrogen content = 12.9% w/w; amino nitrogen = 6.2% w/w; Merck Life Science) in 50 mL of distilled water. Peptone was used as nitrogen source because in previous experiments it was noticed that some *Acinetobacter* and *Rosenbergiella* isolates scarcely grew when alternative sources (e.g., casamino acids, which is predominantly a mixture of free amino acids) was used as nitrogen source [Álvarez-Pérez S., personal observation]. Heating of these sugar + peptone mixtures (50ºC for *c.* 30 min) was required to completely dissolve the sugars. Subsequently, the artificial nectars were sterilized by filtration through 0.22-μm pore diameter syringe filters (VWR, Oud-Heverlee, Belgium) and immediately added into the test plates (96-well plates; BRAND GmbH + Co KG, Wertheim, Germany).

**Preparation of cell suspensions**

Suspensions of *Acinetobacter* and *Rosenbergiella* cells were prepared by scraping bacterial colonies from 96-h old cultures of isolates on trypticase soy agar (TSA; Merck Life Science) with a disposable loop (VWR) and resuspending them in 10 mL of sterile saline solution (0.85% w/v NaCl; Merck Life Science) to reach a transmittance of 85%, as determined using a benchtop turbidimeter (Biolog, Hayward, CA, USA). Typically, this corresponds to a colony forming unit (CFU) count of 1.3·10^7^ ± 1.1·10^7^ CFU/mL for *Acinetobacter* spp. [5] and of 3.4·10^7^ ± 2·10^7^ CFU/mL for *Rosenbergiella* spp. [Álvarez-Pérez S., unpublished results]. Then, bacterial cells were starved by incubating the cell suspensions inside an orbital shaker (150 rpm) at 25°C for 4 h [5].

**Phylogenetic analysis of housekeeping gene sequences**

Nucleotide sequences obtained in previous studies for the *Acinetobacter* and *Rosenbergiella* isolates analyzed in the present study were downloaded from GenBank using R v.4.1.0 [6] and the read.GenBank() function of the R library ‘seqinr’ v.4.2-8 [7] (accession numbers are shown in Tables S1 and S2). Sequences were included in multiple alignments generated by MUSCLE [8] and the resulting alignments were trimmed with BioEdit v.7.0.9.0 [9] to ensure that all sequences had the same start and end point. Sequences differing in at least one nucleotide were classified into different sequence types (STs) using the dereplicate() function of the R library ‘insect’ v.1.4.0.9000 [10]. Maximum likelihood (ML) trees of isolates and STs were built for the *rpoB* gene sequences of *Acinetobacter* and a concatenation of *atpD* + *gyrB* + *rpoB* sequences of *Rosenbergiella* using PhyML v.3.0 [11] with smart model selection based on the Akaike information criterion (AIC) [12]. Branch support was assessed by 1,000 bootstrap resamples and sequences of *Acinetobacter calcoaceticus* NIPH 2245^T^ and *Phaseolibacter flectens* ATCC 12775^T^ were used as outgroups in the ML trees built from *Acinetobacter* and *Rosenbergiella* sequences, respectively. The type strain of *R. nectarea* (8N4^T^) [13] was included in the phylogenetic analyses carried out for *Rosenbergiella*. The resulting trees were visualized and edited with the Molecular Evolutionary Genetics Analysis v.11 (MEGA11) software [14].

**Phylogenomic analysis**

The genome assemblies of the type strains of *Acinetobacter boissieri* (ANC 4422^T^, accession no. GCF_900096955.1), *Acinetobacter nectaris* (CIP 110549^T^, GCF_000488215.1), and *Rosenbergiella nectarea* (8N4^T^, GCF_900111105.1) previously obtained by other authors [15, 16], and the assemblies recently obtained by us for the type strains of *Rosenbergiella australiborealis* (CdVSA20.1^T^ = S264^T^, GCF_018494035.1), *Rosenbergiella collisarenosi* (8.8A^T^ = S260^T^, GCF_018494085.1), and *Rosenbergiella epipactidis* (2.1A^T^ = S256^T^, GCF_018494055.1), and the potentially new species pending of validation *‘Rosenbergiella gaditana’* (S61^T^, GCF_018494065.1) and *‘Rosenbergiella metrosideri’* (JB07^T^, GCF_022602565.1) [4] were retrieved from the NCBI database using Entrez Direct (EDirect) [17]. An ML tree was then built using the up-to-date bacterial core gene set (UBCG2) [18] to extract and align 81 single-copy core genes that are present in most bacterial species known to date (see details in <http://leb.snu.ac.kr/ubcg2/genes/>, accessed on 24 May 2022) and RAxML [19] for phylogeny reconstruction. The genome assembly of *Magnetococcus marinus* MC-1^T^ (Alphaproteobacteria, GCF_000014865.1) was used as outgroup in this analysis. Estimation of the robustness of the nodes in the phylogenomic tree was done through the gene support index (GSI), defined as the number of individual gene trees that present the same node [18]. The consensus tree was visualized and edited with MEGA11.

**Evolutionary model fitting**

The fitContinuous() function of the R library ‘geiger’ v.2.0.7 [20] was used to determine which model of trait evolution provided the best fit to the phenotypic data obtained for *Acinetobacter* and *Rosenbergiella* isolates, STs, and species. The following nine models were tested [20]:

1. Brownian motion [21]: this model assumes that the correlation structure among the trait values of a species pair is proportional to the extent of their shared ancestry.
2. Ornstein-Uhlenbeck [22, 23]: this model incorporates both selection and drift and, therefore, it is more general than pure drift models based on Brownian motion. The Ornstein-Uhlenbeck model assumes random walk with a central tendency proportional to a parameter alpha.
3. Early-burst [24]: this is a model where the rate of evolution increases or decreases exponentially through time.
4. Trend: diffusion model with linear trend in evolutionary rates (toward larger or smaller values) through time.
5. Pagel’s λ [25]: this model multiplies all internal branches of the phylogenetic tree by a parameter λ to fit the extent to which the phylogeny predicts covariance among trait values for species, and leaves tip branches as their original length. Values of lambda close to zero cause the phylogeny to become more star-like, whereas λ = 1 corresponds to pure Brownian motion.
6. Pagel’s κ [25]: punctuational model of trait evolution in which character divergence is related to the number of speciation events between two species and which raises all branch lengths to a power κ. As κ approaches zero, the model becomes speciational, whereas κ = 1 corresponds to pure Brownian motion
7. Pagel’s δ [25]: time-dependent model of trait evolution which raises all node depths to a power δ. When δ < 1, evolution is concentrated early in the tree (i.e., recent evolution has been comparatively slow), whereas δ > 1 concentrates evolution towards the tips (i.e., recent evolution has been relatively fast).
8. Drift: this model of trait evolution considers a directional trend component (toward smaller or larger values through time) or “drift”.
9. White noise model: non-phylogenetic model which assumes that trait values follow a random normal distribution and species have no significant trait covariance.

Interpretation of results and model selection was performed as described in Materials and Methods (see the main text).

**REFERENCES**

1. Álvarez-Pérez S, Lievens B, Jacquemyn H, Herrera CM (2013) *Acinetobacter nectaris* sp. nov. and *Acinetobacter boissieri* sp. nov., isolated from floral nectar of wild Mediterranean insect-pollinated plants. Int J Syst Evol Microbiol 63(4):1532–1539. doi:10.1099/ijs.0.043489-0.
2. Álvarez-Pérez S, Baker LJ, Morris MM, Tsuji K, Sanchez VA, Fukami T, Vannette RL, Lievens B, Hendry TA (2021) *Acinetobacter pollinis* sp. nov., *Acinetobacter baretiae* sp. nov. and *Acinetobacter rathckeae* sp. nov., isolated from floral nectar and honey bees. Int J Syst Evol Microbiol 71(5). doi:10.1099/ijsem.0.004783.
3. Lenaerts M, Alvarez-Pérez S, de Vega C, Van Assche A, Johnson SD, Willems KA, Herrera CM, Jacquemyn H, Lievens B (2014) *Rosenbergiella australoborealis* sp. nov., *Rosenbergiella collisarenosi* sp. nov. and *Rosenbergiella epipactidis* sp. nov., three novel bacterial species isolated from floral nectar. Syst Appl Microbiol 37(6):402–411. doi:10.1016/j.syapm.2014.03.002.
4. Álvarez-Pérez S, de Vega C, Vanoirbeek K, Tsuji K, Jacquemyn H, Fukami T, Michiels C, Lievens B (2021) Phylogenomic analysis of the genus *Rosenbergiella* and description of *Rosenbergiella gaditana* sp. nov., and *Rosenbergiella metrosideri* sp. nov., isolated from floral nectar. Int J Syst Evol Microbiol, submitted.
5. Álvarez-Pérez S, Tsuji K, Donald M, Van Assche A, Vannette RL, Herrera CM, Jacquemyn H, Fukami T, Lievens B (2021) Nitrogen assimilation varies among clades of nectar- and insect-associated acinetobacters. Microb Ecol 81(4):990–1003. doi:10.1007/s00248-020-01671-x.
6. R Core Team (2021). R: A language and environment for statistical computing. R Foundation for Statistical Computing, Vienna, Austria. <https://www.r-project.org/>. Accessed 24 May 2022.
7. Charif D, Lobry JR (2007) SeqinR 1.0-2: a contributed package to the R project for statistical computing devoted to biological sequences retrieval and analysis. In: Bastolla U, Porto M, Roman HE, Vendruscolo M (eds.) Structural Approaches to Sequence Evolution. Biological and Medical Physics, Biomedical Engineering. Springer, Berlin, Heidelberg, pp. 207–232. doi:10.1007/978-3-540-35306-5_10.
8. Edgar RC (2004) MUSCLE: multiple sequence alignment with high accuracy and high throughput. Nucleic Acids Res 32(5):1792–1797. doi:10.1093/nar/gkh340.
9. Hall TA (1999) BioEdit: a user-friendly biological sequence alignment editor and analysis program for Windows 95/98/NT. Nucl Acids Symp Ser 41:95–98.
10. Wilkinson SP, Davy SK, Bunce M, Stat M (2018) Taxonomic identification of environmental DNA with informatic sequence classification trees. PeerJ Preprints 6:e26812v1. doi:10.7287/peerj.preprints.26812v1.
11. Guindon S, Dufayard JF, Lefort V, Anisimova M, Hordijk W, Gascuel O (2010) New algorithms and methods to estimate maximum-likelihood phylogenies: assessing the performance of PhyML 3.0. Syst Biol 59(3):307–321. doi:10.1093/sysbio/syq010.
12. Lefort V, Longueville JE, Gascuel O (2017) SMS: Smart Model Selection in PhyML. Mol Biol Evol 34(9):2422–2424. doi:10.1093/molbev/msx149.
13. Halpern M, Fridman S, Atamna-Ismaeel N, Izhaki I (2013) *Rosenbergiella nectarea* gen. nov., sp. nov., in the family *Enterobacteriaceae*, isolated from floral nectar. Int J Syst Evol Microbiol 63(11):4259–4265. doi:10.1099/ijs.0.052217-0.
14. Tamura K, Stecher G, Kumar S (2021) MEGA11: Molecular Evolutionary Genetics Analysis Version 11. Mol Biol Evol 38(7):3022–3027. doi:10.1093/molbev/msab120.
15. Touchon M, Cury J, Yoon EJ, Krizova L, Cerqueira GC, Murphy C, Feldgarden M, Wortman J, Clermont D, Lambert T, Grillot-Courvalin C, Nemec A, Courvalin P, Rocha EP (2014) The genomic diversification of the whole *Acinetobacter* genus: origins, mechanisms, and consequences. Genome Biol Evol 6(10):2866–2882. doi:10.1093/gbe/evu225.
16. Laviad-Shitrit S, Izhaki I, Whitman WB, Shapiro N, Woyke T, Kyrpides NC, Halpern M (2020) Draft genome of *Rosenbergiella nectarea* strain 8N4^T^ provides insights into the potential role of this species in its plant host. PeerJ 8:e8822. doi:10.7717/peerj.8822.
17. Kans J (2022) Entrez Direct: E-utilities on the Unix Command Line. National Center for Biotechnology Information, Bethesda, USA. <https://www.ncbi.nlm.nih.gov/books/NBK179288/>. Accessed on 24 May 2022.
18. Kim J, Na SI, Kim D, Chun J (2021) UBCG2: Up-to-date bacterial core genes and pipeline for phylogenomic analysis. J Microbiol 59(6):609–615. doi:10.1007/s12275-021-1231-4.
19. Stamatakis A (2014) RAxML version 8: a tool for phylogenetic analysis and post-analysis of large phylogenies. Bioinformatics 30(9):1312–1313. doi:10.1093/bioinformatics/btu033.
20. Pennell MW, Eastman JM, Slater GJ, Brown JW, Uyeda JC, FitzJohn RG, Alfaro ME, Harmon LJ (2014) geiger v2.0: an expanded suite of methods for fitting macroevolutionary models to phylogenetic trees. Bioinformatics 30(15):2216–2218. doi:10.1093/bioinformatics/btu181.
21. Felsenstein J (1973) Maximum-likelihood estimation of evolutionary trees from continuous characters. Am J Hum Genet 25(5):471–492.
22. Butler MA, King AA (2004) Phylogenetic comparative analysis: a modeling approach for adaptive evolution. Am Nat 164(6):683–695. doi:10.1086/426002.
23. Hansen TF (1997) Stabilizing selection and the comparative analysis of adaptation. Evolution 51(5):1341–1351. doi:10.1111/j.1558-5646.1997.tb01457.x.
24. Harmon LJ, Losos JB, Davies TJ, Gillespie RG, Gittleman JL, Jennings WB, Kozak KH, McPeek MA, Moreno-Roark F, Near TJ, Purvis A, Ricklefs RE, Schluter D, Schulte JA, Seehausen O, Sidlauskas BL, Torres-Carvajal O, Weir JT, Mooers AØ (2010) Early bursts of body size and shape evolution are rare in comparative data. Evolution 64(8):2385–2396. doi:10.1111/j.1558-5646.2010.01025.x.
25. Pagel M (1999) Inferring the historical patterns of biological evolution. Nature 401:877–884. doi:10.1038/44766.
