# Supplementary material for: Sugar Concentration, Nitrogen Availability, and Phylogenetic Factors Determine the Ability of Acinetobacter spp. and Rosenbergiella spp. to Grow in Floral Nectar
Source: Microb Ecol. 2022 Aug 5;86(1):377–91. doi: 10.1007/s00248-022-02088-4 (PMC10293439; doi:10.1007/s00248-022-02088-4)
Supplement: Supplementary file 2 — Supplementary file2 (PDF 337 KB) [file 248_2022_2088_MOESM2_ESM.pdf]

SUPPLEMENTARY TABLES

Table S1. Details of the *Acinetobacter* isolates characterized in this study (n = 43).

**Species**[a]	**Isolate name**	**Origin**	**Host organism**	**Sampling location**	**Year of isolation**	**Isolate donor(s)**[b]	***rpoB* sequence type**	***rpoB* sequence (GenBank accession no.)**
A. boissieri	S14	Floral nectar	*Erophaca baetica* (Fabaceae)	Hinojos, Huelva, Spain	2011	SAP	ST15A	JQ771150
	S18	Floral nectar	*Anchusa calcarea* (Boraginaceae)	Hinojos, Huelva, Spain	2011	SAP	ST17A	MN315332
	S21	Floral nectar	*Salvia rosmarinus* (Lamiaceae)	Hinojos, Huelva, Spain	2011	SAP	ST17A	JQ771153
	S27	Floral nectar	*Fritillaria lusitanica* (Liliaceae)	Hinojos, Huelva, Spain	2011	SAP	ST14A	MN315333
	S30	Floral nectar	*Erophaca baetica* (Fabaceae)	Hinojos, Huelva, Spain	2011	SAP	ST16A	MN315336
	S31	Floral nectar	*Lavandula stoechas* (Lamiaceae)	Hinojos, Huelva, Spain	2011	SAP	ST17A	JQ771156
	S318	Floral nectar	*Salvia rosmarinus* (Lamiaceae)	Hinojos, Huelva, Spain	2011	SAP	ST18A	JQ771151
	S319	Floral nectar	*Salvia rosmarinus* (Lamiaceae)	Hinojos, Huelva, Spain	2011	SAP	ST18A	JQ771152
A. nectaris	B3-04	Honeybee (mouth)	*Apis mellifera* (Hymenoptera)	Stanford campus, California, USA	2018	TF, SAP	ST08A	MN315296
	B3-05	Honeybee (mouth)	*Apis mellifera* (Hymenoptera)	Stanford campus, California, USA	2018	TF, SAP	ST08A	MN315297
	B3-06	Honeybee (mouth)	*Apis mellifera* (Hymenoptera)	Stanford campus, California, USA	2018	TF, SAP	ST08A	MN315298
	B3-09	Honeybee (mouth)	*Apis mellifera* (Hymenoptera)	Stanford campus, California, USA	2018	TF, SAP	ST04A	MN315301

B3-10	Honeybee (crop)	*Apis mellifera* (Hymenoptera)	Stanford campus, California, USA	2018	TF, SAP	ST08A	MN315302
B3-11	Honeybee (crop)	*Apis mellifera* (Hymenoptera)	Stanford campus, California, USA	2018	TF, SAP	ST08A	MN315303
B3-12	Honeybee (crop)	*Apis mellifera* (Hymenoptera)	Stanford campus, California, USA	2018	TF, SAP	ST09A	MN315304
B3-13	Honeybee (crop)	*Apis mellifera* (Hymenoptera)	Stanford campus, California, USA	2018	TF, SAP	ST09A	MN315305
B3-16	Honeybee (crop)	*Apis mellifera* (Hymenoptera)	Stanford campus, California, USA	2018	TF, SAP	ST09A	MN315307
B3-17	Honeybee (crop)	*Apis mellifera* (Hymenoptera)	Stanford campus, California, USA	2018	TF, SAP	ST08A	MN315308
EC30	Floral nectar	*Epilobium canum* (Onagraceae)	UC Davis campus, California, USA	2015	RV, MM	ST12A	MN315311
EC31	Floral nectar	*Epilobium canum* (Onagraceae)	UC Davis campus, California, USA	2015	RV, MM	ST06A	MN315312
EC32	Floral nectar	*Epilobium canum* (Onagraceae)	UC Davis campus, California, USA	2015	RV, MM	ST12A	MN315313
EC33	Floral nectar	*Epilobium canum* (Onagraceae)	UC Davis campus, California, USA	2015	RV, MM	ST06A	MN315314
EC43	Floral nectar	*Epilobium canum* (Onagraceae)	UC Davis campus, California, USA	2015	RV, MM	ST05A	MN315317
EC46	Floral nectar	*Epilobium canum* (Onagraceae)	UC Davis campus, California, USA	2015	RV, MM	ST05A	MN315320
EC47	Floral nectar	*Epilobium canum* (Onagraceae)	UC Davis campus, California, USA	2015	RV, MM	ST12A	MN315321
S165	Floral nectar	*Iris xiphium* (Iridaceae)	Hinojos, Huelva, Spain	2011	SAP	ST13A	MN315331
S301	Floral nectar	*Buddleja davidii* (Scrophulariaceae)	Mechelen, Antwerp province, Belgium	2017	SAP	ST03A	MN315337

S302	Floral nectar	*Buddleja davidii* (Scrophulariaceae)	Mechelen, Antwerp province, Belgium	2017	SAP	ST02A	MN315338
S304	Floral nectar	*Buddleja davidii* (Scrophulariaceae)	Mechelen, Antwerp province, Belgium	2017	SAP	ST03A	MN315339
S306	Floral nectar	*Buddleja davidii* (Scrophulariaceae)	Mechelen, Antwerp province, Belgium	2017	SAP	ST02A	MN315340
S313	Floral nectar	*Erophaca baetica* (Fabaceae)	Hinojos, Huelva, Spain	2011	SAP	ST10A	JQ771145
S315	Floral nectar	*Gladiolus illyricus* (Iridaceae)	Hinojos, Huelva, Spain	2011	SAP	ST11A	JQ771147
S316	Floral nectar	*Lonicera implexa* (Caprifoliaceae)	Hinojos, Huelva, Spain	2011	SAP	ST01A	JQ771148
S317	Floral nectar	*Lonicera implexa* (Caprifoliaceae)	Hinojos, Huelva, Spain	2011	SAP	ST01A	JQ771149
S325	Floral nectar	*Linaria vulgaris* (Plantaginaceae)	Mechelen, Antwerp province, Belgium	2017	SAP	ST07A	MN315341
S329	Floral nectar	*Buddleja davidii* (Scrophulariaceae)	Mechelen, Antwerp province, Belgium	2017	SAP	ST02A	MN315342
S330	Floral nectar	*Buddleja davidii* (Scrophulariaceae)	Mechelen, Antwerp province, Belgium	2017	SAP	ST02A	MN315343
S331	Floral nectar	*Buddleja davidii* (Scrophulariaceae)	Mechelen, Antwerp province, Belgium	2017	SAP	ST02A	MN315344
S332	Floral nectar	*Buddleja davidii* (Scrophulariaceae)	Mechelen, Antwerp province, Belgium	2017	SAP	ST02A	MN315345
S333	Floral nectar	*Buddleja davidii* (Scrophulariaceae)	Mechelen, Antwerp province, Belgium	2017	SAP	ST02A	MN315346
S334	Floral nectar	*Buddleja davidii* (Scrophulariaceae)	Mechelen, Antwerp province, Belgium	2017	SAP	ST02A	MN315347
S335	Floral nectar	*Buddleja davidii* (Scrophulariaceae)	Mechelen, Antwerp province, Belgium	2017	SAP	ST02A	MN315348

S336	Floral nectar	*Buddleja davidii* (Scrophulariaceae)	Mechelen, Antwerp province, Belgium	2017	SAP	ST02A	MN315349

[a] As determined by BLASTn searches of *rpoB* sequences against the GenBank databases.

[b] Isolate donors: MM, Megan Morris (Stanford University, USA); RV, Rachel Vannette (UC Davis, USA); SAP, Sergio Álvarez-Pérez (Complutense University of Madrid, Spain); TF, Tadashi Fukami (Stanford University, USA). Tryptone soy agar was used for the isolation and subculturing of all isolates.

Table S2. Details of the *Rosenbergiella* isolates characterized in this study (*n* = 45).

**Species**[a]	**Isolate name**[b]	**Origin**	**Host organism**	**Sampling location**	**Year of isolation**	**Isolate donor(s)**[c]	**Sequence type**	**GenBank accession numbers**[d]
R. australiborealis	S264[T]	Floral nectar	*Protea roupelliae* (Proteaceae)	Mount Gilboa, KwaZulu-Natal, South Africa	2011	CdV, SAP	ST23R	KF876198, KF876208, KF876215
	S265	Floral nectar	*Protea roupelliae* (Proteaceae)	Mount Gilboa, KwaZulu-Natal, South Africa	2011	CdV, SAP	ST23R	KF876201, KF876209, KF876216
	S266	Floral nectar	*Protea subvestita* (Proteaceae)	Sani Pass, KwaZulu-Natal, South Africa	2011	CdV, SAP	ST24R	KF876199, KF876210, KF876217
R. collisarenosi	S260[T]	Floral nectar	*Epipactis palustris* (Orchidaceae)	Ter Yde, Oostduinkerke, West Flanders, Belgium	2012	BL, HJ	ST22R	KF876193, KF876202, KF876214
	JB25	Floral nectar	*Metrosideros polymorpha* (Myrtaceae)	Hawai'i Volcanoes National Park, Hawaii, USA	2013	RRJ	ST19R	MT354630, MT354669, MT354708
	S147	Floral nectar	*Phlomis purpurea* (Lamiaceae)	P. N. S[a] Hornachuelos, Córdoba, Spain	2011	SAP	ST21R	MT354633, MT354672, MT354711
	S294	Floral nectar	*Buddleja davidii* (Scrophulariaceae)	Mechelen, Antwerp province, Belgium	2017	SAP	ST20R	MT354634, MT354673, MT354712
	S7	Floral nectar	*Narcissus papyraceus* (Amaryllidaceae)	Hinojos, Huelva, Spain	2011	SAP	ST19R	MT354631, MT354670, MT354709
	S99	Floral nectar	*Iris xiphium* (Iridaceae)	Hinojos, Huelva, Spain	2011	SAP	ST18R	MT354632, MT354671, MT354710

R. epipactidis	S256^{T}	Floral nectar	*Epipactis palustris* (Orchidaceae)	Dune du Perroquet, Bray-Dunes, France	2012	BL, HJ	ST03R	KF876195, KF876204, KF876212
	FR72	Floral nectar	*Diplacus (Mimulus) aurantiacus* (Phrymaceae)	Jasper Ridge Biological Preserve, Stanford, California, USA	2017	KT, TF	ST10R	MT354639, MT354678, MT354717
	JB02	Floral nectar	*Metrosideros polymorpha* (Myrtaceae)	Hawai'i Volcanoes National Park, Hawaii, USA	2013	RRJ	ST09R	MT354640, MT354679, MT354718
	JB21	Floral nectar	*Metrosideros polymorpha* (Myrtaceae)	Hawai'i Volcanoes National Park, Hawaii, USA	2013	RRJ	ST07R	MT354642, MT354681, MT354720
	JR114	Floral nectar	*Diplacus (Mimulus) aurantiacus* (Phrymaceae)	Jasper Ridge Biological Preserve, Stanford, California, USA	2018	TF, SAP	ST10R	MT354643, MT354682, MT354721
	K1916	Floral nectar	*Diplacus (Mimulus) aurantiacus* (Phrymaceae)	Jasper Ridge Biological Preserve, Stanford, California, USA	2017	KT, TF	ST06R	MT354644, MT354683, MT354722
	K24	Floral nectar	*Eurya japonica* (Pentaphylacaceae)	Takaike Kozagawacho Higashimurogun, Wakayama prefecture, Japan	2016	KT, TF	ST11R	MT354645, MT354684, MT354723
	K264	Floral nectar	*Diplacus (Mimulus) aurantiacus* (Phrymaceae)	Jasper Ridge Biological Preserve, Stanford, California, USA	2017	KT, TF	ST05R	MT354646, MT354685, MT354724
	K265	Floral nectar	*Diplacus (Mimulus) aurantiacus* (Phrymaceae)	Jasper Ridge Biological Preserve, Stanford, California, USA	2017	KT, TF	ST05R	MT354647, MT354686, MT354725
	K371	Floral nectar	*Diplacus (Mimulus) aurantiacus* (Phrymaceae)	Jasper Ridge Biological Preserve, Stanford, California, USA	2017	KT, TF	ST06R	MT354648, MT354687, MT354726

	K372	Floral nectar	*Diplacus (Mimulus) aurantiacus* (Phrymaceae)	Jasper Ridge Biological Preserve, Stanford, California, USA	2017	KT, TF	ST06R	MT354649, MT354688, MT354727
	S50	Floral nectar	*Antirrhinum* sp. (Plantaginaceae)	Barbate, Cádiz, Spain	2011	SAP	ST04R	MT354650, MT354689, MT354728
	S55	Floral nectar	*Antirrhinum* sp. (Plantaginaceae)	Barbate, Cádiz, Spain	2011	SAP	ST10R	MT354651, MT354690, MT354729
	S67	Floral nectar	*Antirrhinum* sp. (Plantaginaceae)	Barbate, Cádiz, Spain	2011	SAP	ST02R	MT354652, MT354691, MT354730
	S68	Floral nectar	*Antirrhinum* sp. (Plantaginaceae)	Barbate, Cádiz, Spain	2011	SAP	ST01R	MT354653, MT354692, MT354731
	S76	Floral nectar	*Lathyrus* sp. (Fabaceae)	Barbate, Cádiz, Spain	2011	SAP	ST01R	MT354654, MT354693, MT354732
'R. gaditana'	S61^{T}	Floral nectar	*Antirrhinum* sp. (Plantaginaceae)	Barbate, Cádiz, Spain	2011	SAP	ST17R	MT354635, MT354674, MT354713
	S284	Floral nectar	*Echium* sp. (Boraginaceae)	Madrid, Spain	2017	SAP	ST17R	MT354636, MT354675, MT354714
	S290	Floral nectar	*Echium* sp. (Boraginaceae)	Madrid, Spain	2017	SAP	ST17R	MT354637, MT354676, MT354715
'R. metrosideri'	JB07	Floral nectar	*Metrosideros polymorpha* (Myrtaceae)	Hawai'i Volcanoes National Park, Hawaii, USA	2013	RRJ	ST08R	MT354641, MT354680, MT354719
R. nectarea	B1A^{T}	Honeybee (mouth)	*Apis mellifera*	Stanford campus, California, USA	2018	TF, SAP	ST14R	MT354655, MT354694, MT354733

B3A	Honeybee (mouth)	*Apis mellifera*	Stanford campus, California, USA	2018	TF, SAP	ST15R	MT354656, MT354695, MT354734
B4A	Bumblebee (gut)	*Bombus* sp.	Stanford campus, California, USA	2018	TF, SAP	ST14R	MT354657, MT354696, MT354735
B5A	Honeybee (mouth)	*Apis mellifera*	Stanford campus, California, USA	2018	TF, SAP	ST14R	MT354658, MT354697, MT354736
FNA5	Floral nectar	*Buddleja davidii* (Scrophulariaceae)	Mechelen (Antwerp, Belgium)	2017	SAP	ST16R	MT354659, MT354698, MT354737
FR67	Floral nectar	*Buddleja davidii* (Scrophulariaceae)	Mechelen (Antwerp, Belgium)	2017	SAP	ST16R	MT354660, MT354699, MT354738
K1039	Floral nectar	*Buddleja davidii* (Scrophulariaceae)	Mechelen (Antwerp, Belgium)	2017	SAP	ST16R	MT354661, MT354700, MT354739
K353	Floral nectar	*Buddleja davidii* (Scrophulariaceae)	Mechelen (Antwerp, Belgium)	2017	SAP	ST16R	MT354662, MT354701, MT354740
M26	Floral nectar	*Erophaca baetica* (Fabaceae)	Hinojos (Huelva, Spain)	2011	SAP	ST16R	MT354663, MT354702, MT354741
S255	Floral nectar	*Epipactis palustris* (Orchidaceae)	Dune Dewulf, Ghyvelde, France	2012	BL, HJ	ST13R	KF876194, KF876203, KF876211
S258	Floral nectar	*Epipactis palustris* (Orchidaceae)	Dune du Perroquet, Bray-Dunes, France	2012	BL, HJ	ST12R	KF876196, KF876205, KF876213
S292	Floral nectar	*Symphytum officinale* (Boraginaceae)	Mechelen, Antwerp province, Belgium	2017	SAP	ST16R	MT354664, MT354703, MT354742

	S321	Floral nectar	*Linaria vulgaris* (Scrophulariaceae)	Sint-Katelijne-Waver, Antwerp province, Belgium	2017	SAP	ST16R	MT354665, MT354704, MT354743
	S32	Floral nectar	*Linaria vulgaris* (Scrophulariaceae)	Sint-Katelijne-Waver, Antwerp province, Belgium	2017	SAP	ST16R	MT354666, MT354705, MT354744
	S324	Floral nectar	*Linaria vulgaris* (Scrophulariaceae)	Sint-Katelijne-Waver, Antwerp province, Belgium	2017	SAP	ST16R	MT354667, MT354706, MT354745
	ST2	Floral nectar	*Linaria vulgaris* (Scrophulariaceae)	Leuven-Heverlee, Flemish Brabant, Belgium	2013	BL, HJ	ST16R	MT354668, MT354707, MT354746

[a] As determined by BLASTn searches of *atpD*, *gyrB*, and *rpoB* sequences against the GenBank databases. Species names pending of validation are indicated between quotation marks.

[b] ^{T}: type strain.

[c] Isolate donors: BL, Bart Lievens (KU Leuven, Belgium), CdV, Clara de Vega (University of Seville, Spain); HJ, Hans Jacquemyn (KU Leuven, Belgium); KT, Kaoru Tsuji (Kobe University, Japan); RRJ, Robert R. Junker (Philipps-University Marburg, Germany); SAP, Sergio Álvarez-Pérez (Complutense University of Madrid, Spain); TF, Tadashi Fukami (Stanford University, California, USA). Tryptone soy agar was used for the isolation and subculturing of all isolates.

[d] Accession numbers for the *atpD*, *gyrB*, and *rpoB* gene sequences (in this order).

Table S3. Overview of the optical density results obtained in the growth assays performed in this study.

Artificial nectar[a]	OD (3 d)[b]	OD (7 d)[b]	Kept for analysis?[c]
snS	$-6\cdot10^{-4}$–0.037	-0.002–0.031	No
sNS	0.038–0.381	0.005–0.489	Yes
SnS	-0.003–0.002	-0.015–0.005	No
SNS	-0.003–0.078	-0.009–0.071	Yes
snM	-0.002–0.021	-0.002–0.021	No
sNM	0.045–0.311	0.067–0.401	Yes
SnM	-0.005–0.002	-0.004–0.002	No
SNM	-0.003–0.013	-0.003–0.027	No
snH	-0.003–0.013	-0.012–0.014	No
sNH	0.043–0.266	0.049–0.393	Yes
SnH	-0.004–0.002	-0.009–0.002	No
SNH	-0.005–0.001	-0.018–0.001	No

[a] Artificial nectar codes are as in Table 1.

[b] Range of average optical densities (OD) obtained after 3 and 7 days of incubation of test plates. See details in the main text.

[c] Tests yielding average OD values < 0.05 after 7 days of incubation for all isolates were not kept for detailed data analysis.

Table S4. Intraplate reproducibility of the growth assays performed in this study.

Incubation time	Artificial nectar[a]	CCC estimate (95% CI)[b]
3 days	sNS	0.965 (0.935-0.981)
	SNS	0.985 (0.971-0.992)
	sNM	0.908 (0.833-0.950)
	sNH	0.775 (0.628-0.869)
7 days	sNS	0.972 (0.948-0.985)
	SNS	0.964 (0.932-0.981)
	sNM	0.835 (0.712-0.909)
	sNH	0.725 (0.553-0.838)

[a] Artificial nectar codes are as in Table 1.

[b] Lin's concordance correlation coefficient (CCC) for agreement on continuous measures (mean estimate and 95% confidence interval). *n* = 40 in all cases.

Table S5. Model fitting results obtained for the growth performance of *Acinetobacter* and *Rosenbergiella* isolates, sequence types (STs), and species in different artificial nectars.

**Phylogenetic tree (*N*)**[a]	**Artificial nectar**[b]	**Evolutionary models**[c]								
		**Brownian motion**	**Pagel's δ**	**Drift**	**Early burst**	**Pagel's κ**	**Pagel's λ**	**Ornstein-Uhlenbeck**	**Trend**	**White noise**
Acinetobacter isolates (45)	SNS	<0.001	<0.001	<0.001	<0.001	0.729*	0.271	<0.001	<0.001	<0.001
	sNH	<0.001	<0.001	<0.001	<0.001	0.307	0.693*	<0.001	<0.001	<0.001
	sNM	<0.001	<0.001	<0.001	<0.001	0.925*	0.074	<0.001	<0.001	0.001
	sNS	<0.001	<0.001	<0.001	<0.001	0.138	0.862*	<0.001	<0.001	<0.001
Acinetobacter STs (18)	SNS	<0.001	<0.001	<0.001	<0.001	0.380	0.618*	<0.001	<0.001	0.002
	sNH	<0.001	<0.001	<0.001	<0.001	0.661*	0.339	<0.001	<0.001	<0.001
	sNM	<0.001	<0.001	<0.001	<0.001	0.900*	0.078	<0.001	<0.001	0.022
	sNS	<0.001	<0.001	<0.001	<0.001	0.136	0.843*	<0.001	<0.001	0.020
Rosenbergiella isolates (47)	SNS	<0.001	<0.001	<0.001	<0.001	0.877*	0.123	<0.001	<0.001	<0.001
	sNH	<0.001	<0.001	<0.001	<0.001	1.000*	<0.001	<0.001	<0.001	<0.001
	sNM	<0.001	<0.001	<0.001	<0.001	1.000*	<0.001	<0.001	<0.001	<0.001
	sNS	<0.001	<0.001	<0.001	<0.001	0.068	0.932*	<0.001	<0.001	<0.001
Rosenbergiella STs (24)	SNS	0.020	0.084	0.007	0.007	0.672*	0.161	0.016	0.028	0.003
	sNH	<0.001	<0.001	<0.001	<0.001	0.187	0.219	<0.001	<0.001	0.594*
	sNM	<0.001	<0.001	<0.001	<0.001	0.020	0.263	<0.001	<0.001	0.716*
	sNS	<0.001	<0.001	<0.001	<0.001	0.035	0.259	<0.001	<0.001	0.705*
Species (8)	SNS†	0.060	0.063	0.022	0.022	0.027	0.170	0.130	0.043	0.462
	sNH	0.037	0.042	0.014	0.014	0.030	0.198	0.100	0.027	0.538*
	sNM	<0.001	<0.001	<0.001	<0.001	0.038	0.258	0.003	<0.001	0.700*
	sNS	0.014	0.017	0.005	0.005	0.044	0.230	0.048	0.011	0.626*

[a] Phylogenetic tree used for evolutionary model testing. *N*: number of isolates, STs, or genomes available for analysis.

[b] Artificial nectar codes are as in Table 1. A dagger after the code means that none of the evolutionary models performed substantially better than the others (i.e., all Akaike weights were <0.5).

[c] Akaike weights obtained for the different models of trait evolution analyzed in this study. Asterisks denote the best model (i.e., the one yielding the greater Akaike weight) for each test condition.
