# Supplementary material for: Sugar Concentration, Nitrogen Availability, and Phylogenetic Factors Determine the Ability of Acinetobacter spp. and Rosenbergiella spp. to Grow in Floral Nectar
Source: Microb Ecol. 2022 Aug 5;86(1):377–91. doi: 10.1007/s00248-022-02088-4 (PMC10293439; doi:10.1007/s00248-022-02088-4)

SUPPLEMENTARY FIGURES

Figure S1. Maximum likelihood consensus trees built from partial *rpoB* gene sequences (861 bp) of the *Acinetobacter* isolates included in this study. GenBank accession numbers are shown in Table S1. Evolutionary distances (number of base substitutions per site) were computed using a general time reversible substitution model with gamma-distributed rate variation across sites (GTR + G model; gamma shape parameter = 0.36, as estimated by PhyML), with four substitution rate categories, starting trees generated by BIONJ, and nearest neighbor interchange tree search algorithm. Bootstrap node support values (based on 1000 simulations) ≥90% are indicated by red numbers at branching points. The *rpoB* gene sequence of *Acinetobacter calcoaceticus* NIPH 2245^{T} (GenBank accession no. EU477149) was used as outgroup and the tree was rooted on the midpoint. The sequence type (ST) of each isolate is indicated between brackets and the species affiliation is shown at the tips of branches (see also Table S1).

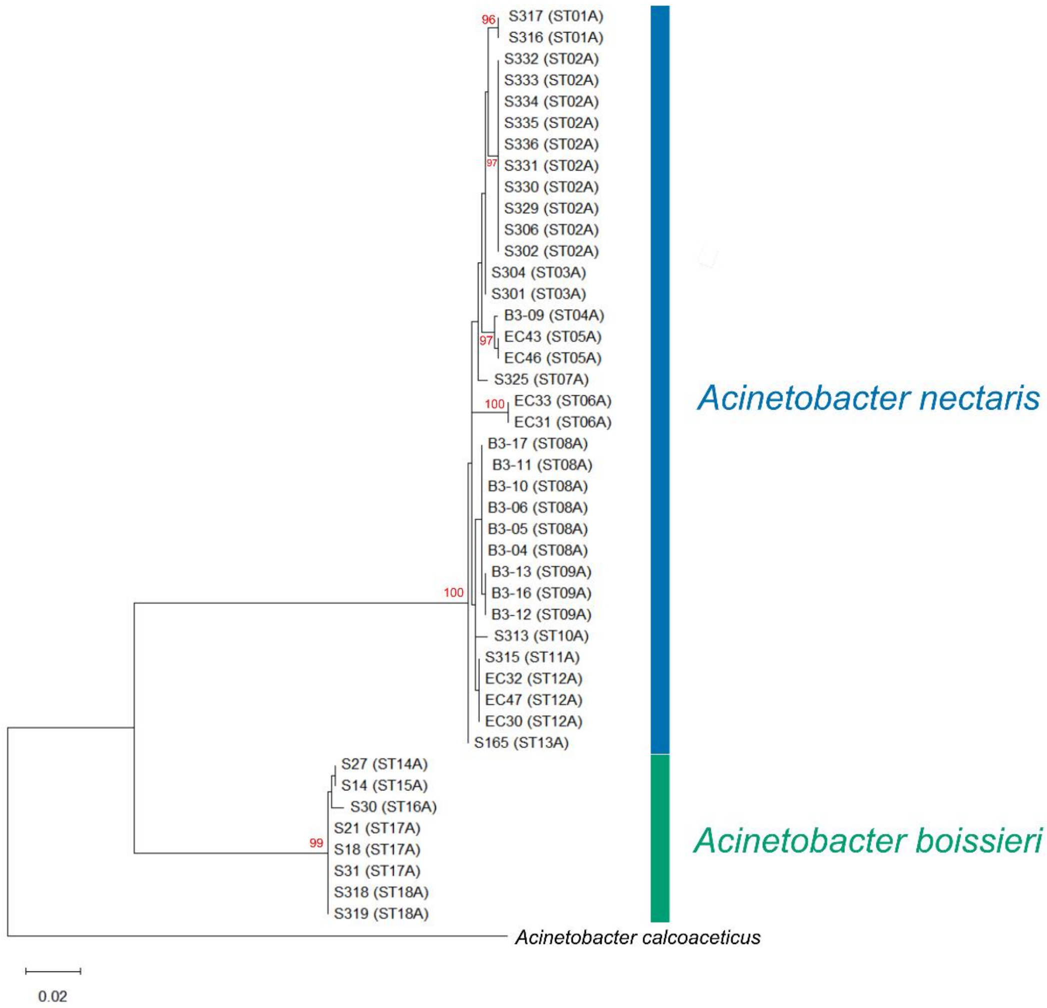

Figure S2. Maximum likelihood consensus trees built from a concatenation of *atpD* + *gyrB* + *rpoB* gene sequences (1863 bp in total) of the *Rosenbergiella* isolates included in this study (*n* = 47). GenBank accession numbers are shown in Table S2. Evolutionary distances (number of base substitutions per site) were computed using a general time reversible substitution model with gamma distributed rate variation among sites and a proportion of invariable sites (GTR + G + I model; gamma shape parameter = 0.45 and proportion of invariant = 0.46, as estimated by PhyML), with four substitution rate categories, starting trees generated by BIONJ, and nearest neighbor interchange tree search algorithm. Bootstrap node support values (based on 1000 simulations) ≥90% are indicated by red numbers at branching points. A concatenation of *atpD*, *gyrB*, and *rpoB* sequences of *Rosenbergiella nectarea* 8N4^T (GenBank accession nos. JN808189, JF745806, and JF745805, respectively) was also included in the phylogenetic analysis. *Phaseolibacter flectens* ATCC 12775^T (GenBank accession nos. JN808190 for *atpD*, JF745803 for *gyrB*, and JF745804 for *rpoB*) was used as outgroup and the tree was rooted on the midpoint. The sequence type (ST) of each isolate is indicated between brackets and the species affiliation is shown at the tips of branches (see also Table S2). Species names pending of validation are indicated between quotation marks.

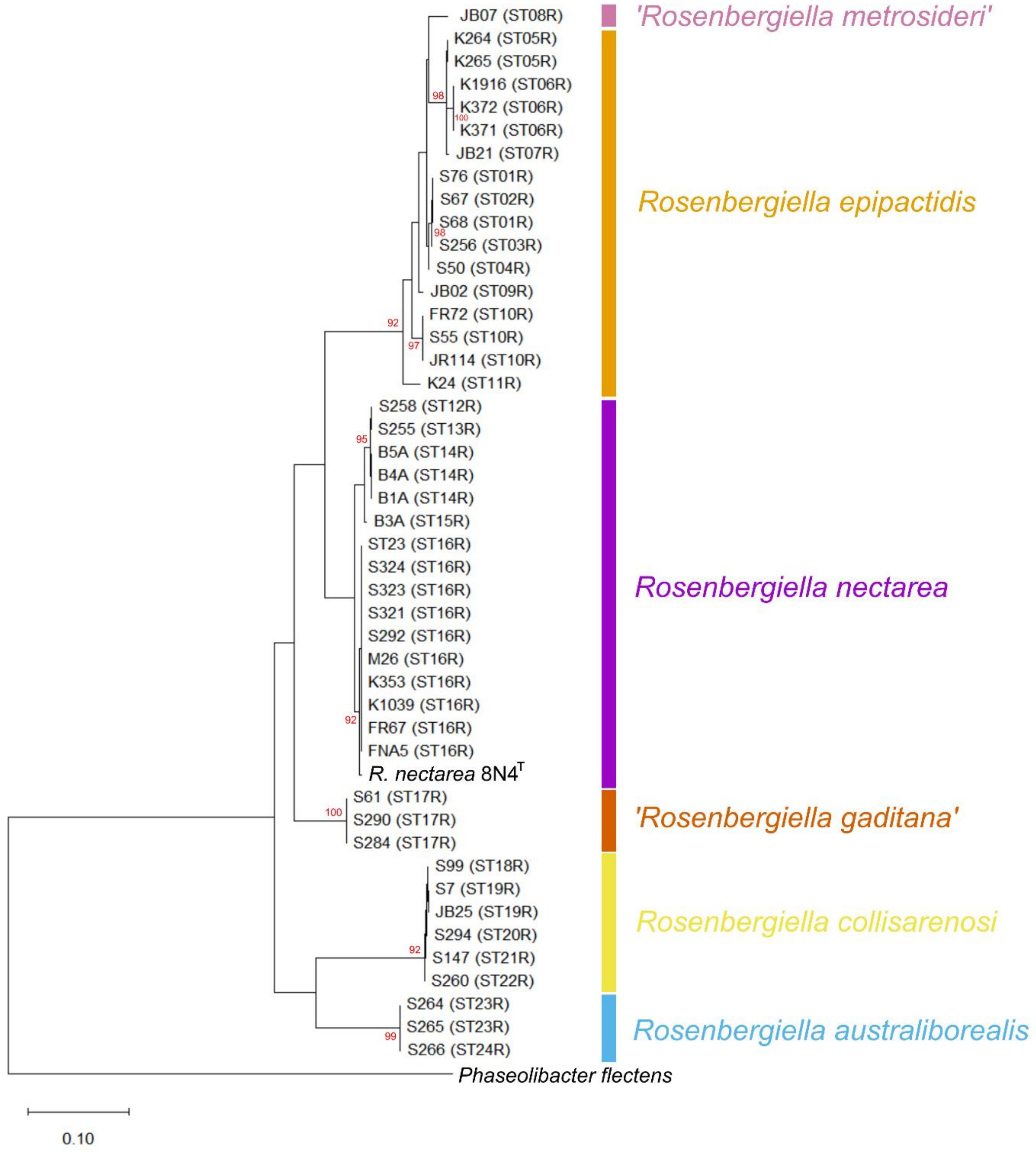

Figure S3. Maximum likelihood phylogenetic tree generated from up-to-date bacterial core gene (UBCG) set showing the relationships between the *Acinetobacter* and *Rosenbergiella* species analyzed in this study (species names pending of validation are indicated between quotation marks). Gene support indices (GSI) are indicated by red numbers at branching points, and *Magnetococcus marinus* MC-1^{T} was used as the outgroup. GenBank accession numbers for genome assemblies used to obtain the tree are provided in 'Materials and methods'. Bar, 0.5 substitutions per nucleotide position.

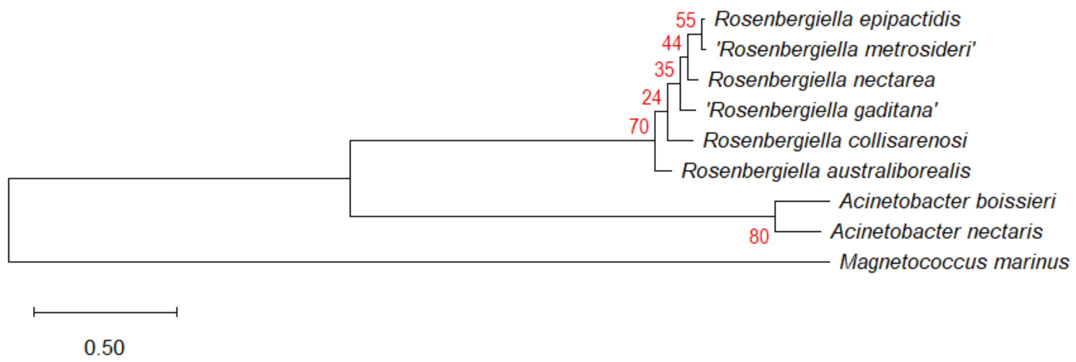

Figure S4. Lin's concordance correlation coefficient plots showing the agreement between intraplate replicates of the growth tests in artificial nectars. The regression line for the intraplate replicates (continuous line) and the line of perfect concordance (y = x; dashed line) are shown in each plot. Artificial nectar codes are as in Table 1 and the number (3 or 7) after these codes denotes the day when the growth results were determined (day 3 or day 7 post-inoculation, respectively).

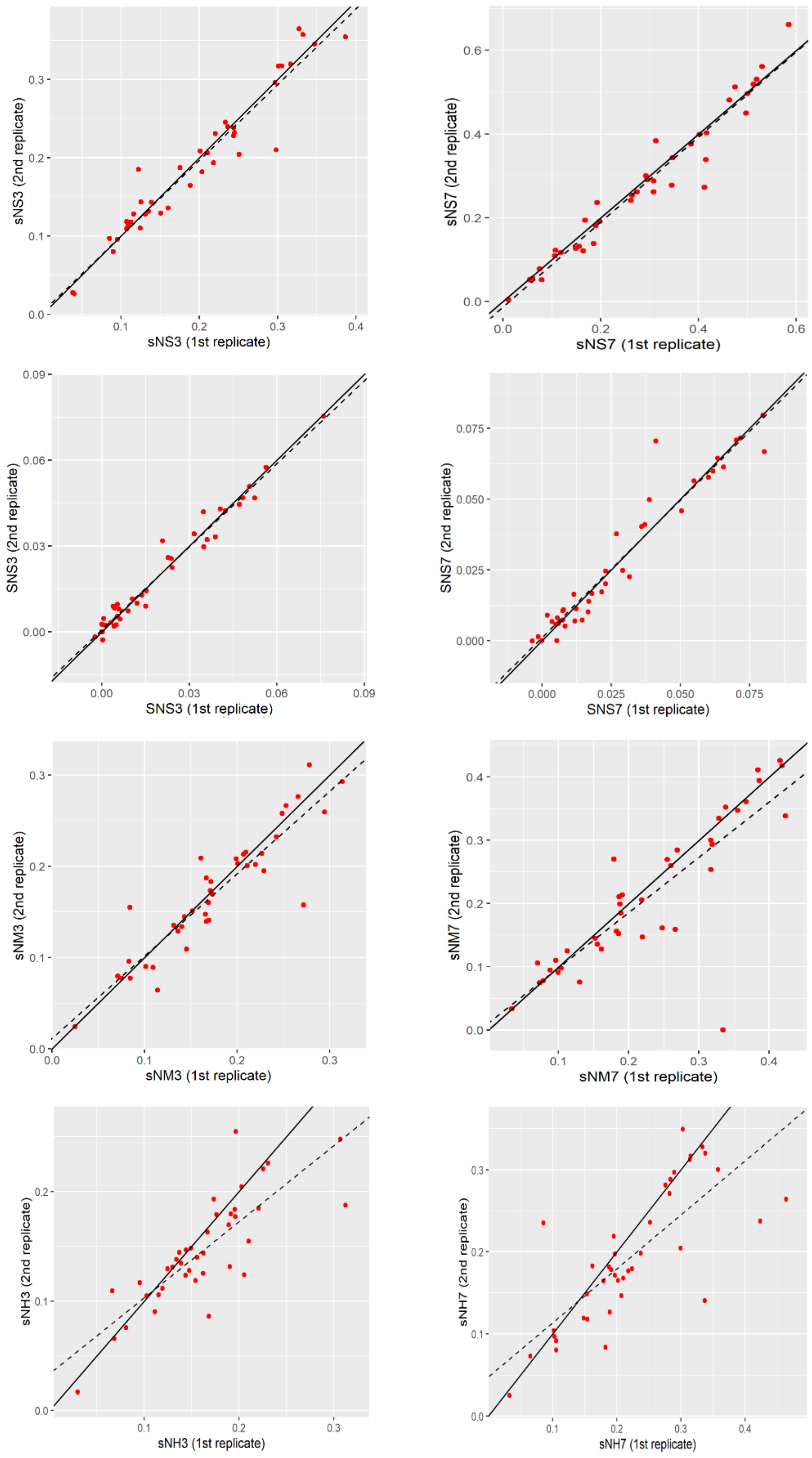

Figure S5. Correlation of the growth data obtained in this study for *Acinetobacter* and *Rosenbergiella* isolates (*left* and *right*, respectively). Colors show the correlation (Spearman's ρ) between the phylogenetically independent contrasts (PICs) obtained for each pair of traits (considering the artificial nectars and incubation times). Significant correlations are shown in the lower triangle. Artificial nectar codes are as in Table 1 and the number (3 or 7) after these codes denotes the day when the growth results were determined (day 3 or day 7 post-inoculation, respectively).

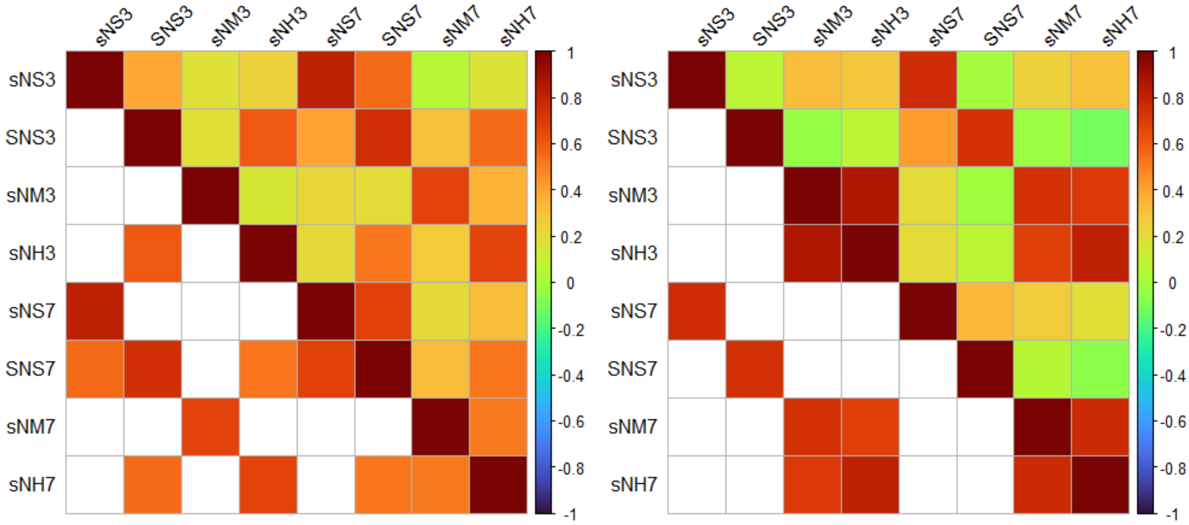

Figure S6. Phylogenetic heatmap of the trait values obtained for the different *Acinetobacter rpoB* sequence types (STs, shown in rows; see Figure S1) in the growth assays performed in this study. Artificial nectar codes (shown in columns) as in Table 1.

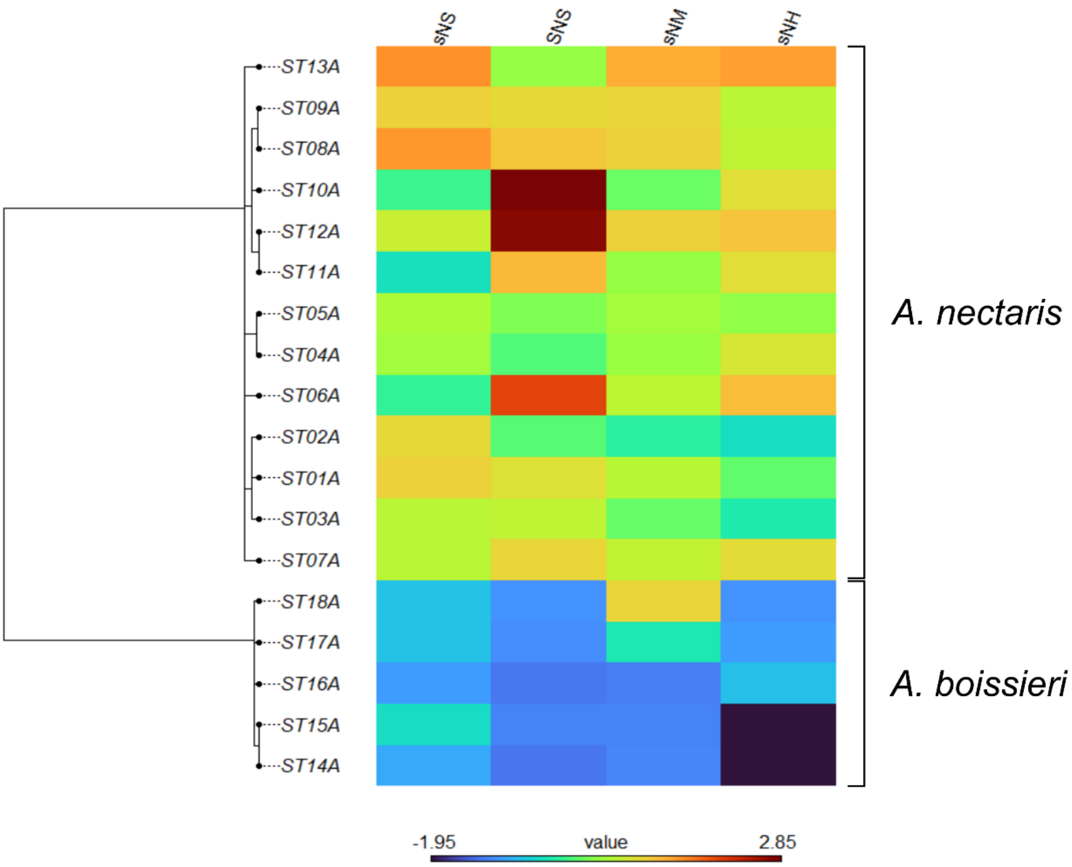

Figure S7. Phylogenetic heatmap of the trait values obtained for the different *Rosenbergiella atpD* + *gyrB* + *rpoB* sequence types (STs, shown in rows; see Figure S2) in the growth assays performed in this study. Artificial nectar codes (shown in columns) are as in Table 1. Species names pending of validation are indicated between quotation marks.

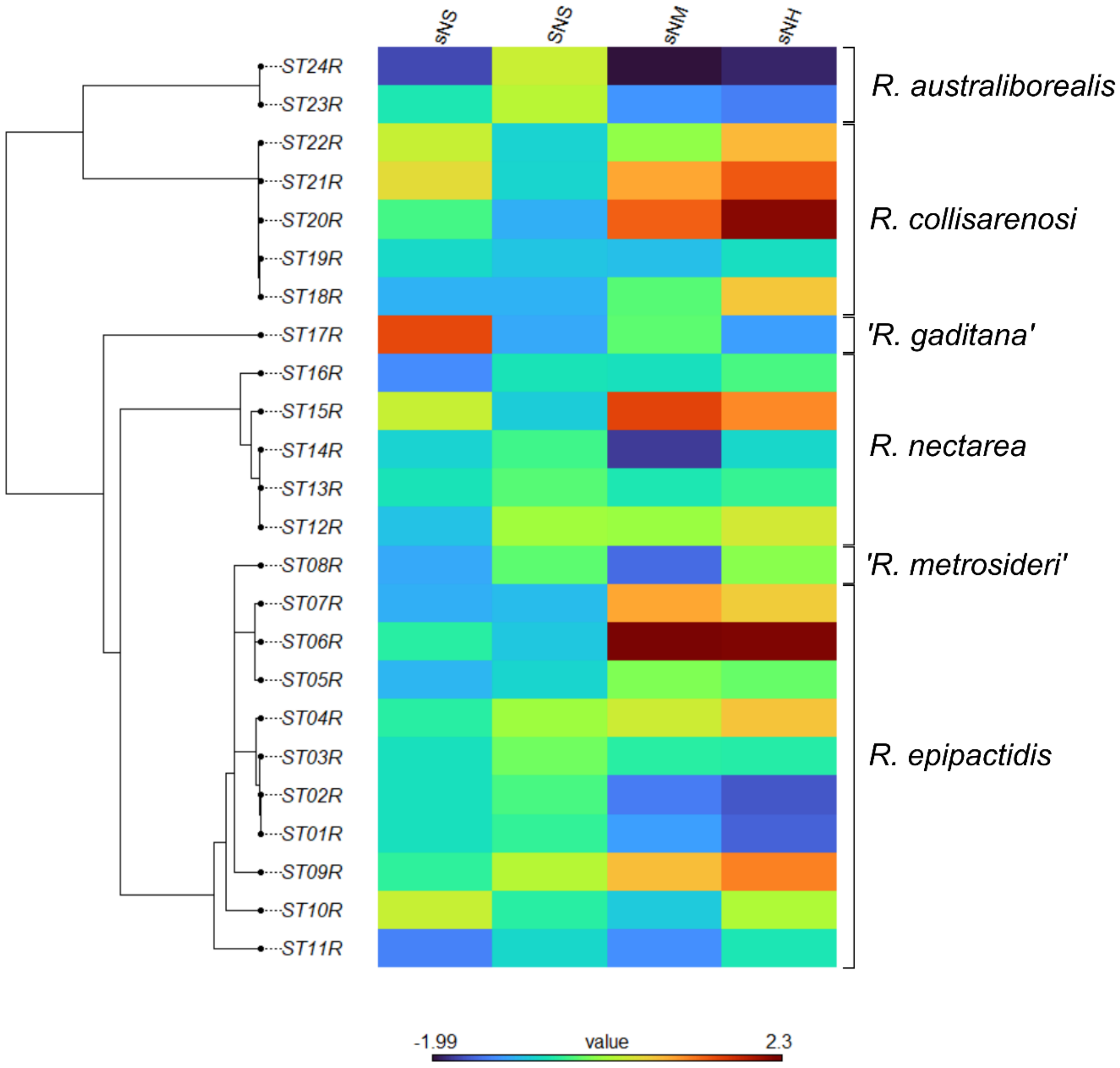

Figure S8. Phylogenetic heatmap of the trait values obtained for different *Acinetobacter* and *Rosenbergiella* species (shown in rows) in the growth assays performed in this study. Artificial nectar codes (shown in columns) are as in Table 1. The phylogenetic tree shown on the left part of the figure was built from whole genome sequences of the studied species (see Figure S3). Abbreviations of species names: AB, *Acinetobacter boissieri*; AN, *Acinetobacter nectaris*; RA, *Rosenbergiella australiborealis*; RC, *Rosenbergiella collisarenosi*; RE, *Rosenbergiella epipactidis*; RG, *'Rosenbergiella gaditana'*; RM, *'Rosenbergiella metrosideri'*; RN, *Rosenbergiella nectarea*.

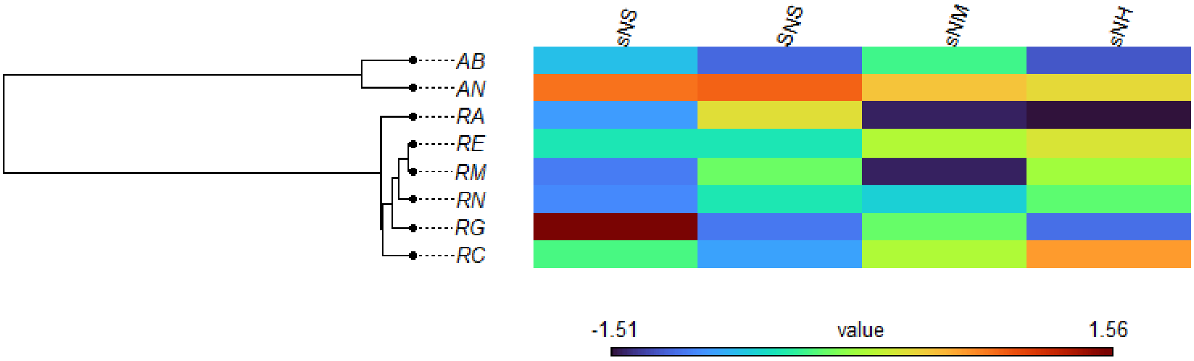

Supplement: Supplementary file 3 — Supplementary file3 (PDF 843 KB) [file 248_2022_2088_MOESM3_ESM.pdf]
